# Comparative transcriptomics reveals small RNA composition and differential microRNA responses underlying interferon-mediated antiviral regulation in porcine alveolar macrophages

**DOI:** 10.3389/fimmu.2022.1016268

**Published:** 2022-10-28

**Authors:** Jiuyi Li, Eric R. Sang, Oluwaseun Adeyemi, Laura C. Miller, Yongming Sang

**Affiliations:** ^1^Department of Agricultural and Environmental Sciences, College of Agriculture, Tennessee State University, Nashville, TN, United States; ^2^USDA, Agricultural Research Service, National Animal Disease Center, Virus and Prion Research Unit, Ames, IA, United States

**Keywords:** small RNA, microRNA, interferon, porcine reproductive and respiratory syndrome virus, porcine alveolar macrophages

## Abstract

Previous studies have shown that interferon-mediated antiviral activity is subtype-dependent. Using a whole transcriptome procedure, we aimed to characterize the small RNA transcriptome (sRNA-Seq) and specifically the differential microRNA (miRNA) responses in porcine alveolar macrophages (PAMs) upon antiviral activation during viral infection and interferon (IFN) stimulation. Data showed that near 90% of the qualified reads of sRNA were miRNAs, and about 10% of the other sRNAs included rRNA, snoRNA, snRNA, and tRNA in order of enrichment. As the majority of sRNA (>98%) were commonly detected in all PAM samples under different treatments, about 2% sRNA were differentially expressed between the different antiviral treatments. Focusing on miRNA, 386 miRNA were profiled, including 331 known and 55 novel miRNA sequences, of which most were ascribed to miRNA families conserved among vertebrates, particularly mammalian species. Of the miRNA profiles comparably generated across the different treatments, in general, significantly differentially expressed miRNA (SEM) demonstrated that: (1) the wild-type and vaccine strains of a porcine arterivirus (a.k.a., PRRSV) induced nearly reversed patterns of up- or down-regulated SEMs; (2) similar SEM patterns were found among the treatments by the vaccine strain and antiviral IFN-α1/-ω5 subtypes; and (3) the weak antiviral IFN-ω1, however, remarked a suppressive SEM pattern as to SEMs upregulated in the antiviral treatments by the vaccine and IFN-α1/-ω5 subtypes. Further articulation identified SEMs commonly or uniquely expressed in different treatments, and experimentally validated that some SEMs including miR-10b and particularly miR-9-1 acted significantly in regulation of differential antiviral reactions stimulated by different IFN subtypes. Therefore, this study provides a general picture of porcine sRNA composition and pinpoints key SEMs underlying antiviral regulation in PAMs correlated to a typical respiratory RNA virus in pigs.

## Introduction

The alveolar macrophage (AM) is a specific tissue macrophage that monitors the alveolar–blood interface and provides a front line of cellular defense against air-borne pollutants and respiratory pathogens (1–4). As innate immune cells, activated AMs secrete host defense peptides, lysozyme, proteases, and active oxygen/nitrogen species; and exploit processes of phagocytosis and intracellular killing to eliminate microbes that are constantly aspirated in mammals. Alveolar macrophages also have crucial roles in immunomodulation of alveolar defenses against respiratory infections (1, 2). While confronting surmounting or virulent microbes, AMs produce metabolic mediators and cytokines/chemokines including interleukin (IL)-1, IL-6, interferons (IFN), IL-8, and tumor necrosis factor (TNF)-α, which are critical in the mediation of inflammatory and immune responses underlying the microbe-host interaction (2, 4). In infections by respiratory viruses, AMs serve as a primary producer of antiviral IFNs (5, 6), which are mostly type I IFNs, including subtypes of IFN-α/-β/-ω, and type III IFNs also known as IFN-λ (7). Consequently, AMs are activated by IFN stimulation by different subtypes and differentiate status pertaining to antiviral or immunomodulatory variance against viral infections (1–3). In the mammalian respiratory tract, respiratory epithelial cells and AMs are the primary cells that encounter the invading viruses. Studies showed that AM depletion, such as in ferrets, mice or pigs, severely dampen protective pro-inflammatory responses, resulting in severe lung lesions, dysregulated inflammation and significant mortality in animals infected by influenza viruses (8–10). Alternately, viruses that evolve a capacity to suppress or evade AM’s surveillance are prone to cause productive infections in the lung. Hence, a plethora of respiratory viruses are AM-tropic to directly infect AMs, impede AM-mediated immune responses, and even hijack AMs to facilitate the virus spreading in the host (3, 11). In this regard, porcine reproductive and respiratory syndrome virus (PRRSV) represents a major respiratory virus, which takes AMs as primary sites of infection causing frequent epizootics and has devastated swine production worldwide for over 30 years (3, 11). Recent studies indicated a correlation between the pathogenicity of PRRSV strains and their capacity to infect AMs as well as to subvert AM’s inflammatory response (11). However, molecular mechanisms underlying this correlation have largely remained unstudied. Given that most marketed vaccines for PRRSV prevention use live-attenuated viruses of relevant pathogenic field isolates, a paralleled comparison of these vaccine strains with their parental viruses will elucidate molecular markers to assist future vaccine design countering the ever-emerging highly-pathogenic PRRSV strains (12).

Comparing whole genome sequences of vertebrates, the domestic swine genome contains the highest number of genes (near 60) that encode seven IFN subtypes including IFN-α/-β/-δ/-ϵ/-κ/-μ/-ω (7, 13). Our previous studies characterized the porcine IFN complex, and demonstrated the tissue-dependent differential expression, as well as the subtype-dependent activity in terms of the induction of IFN-stimulated genes (ISGs), anti-proliferation on cells, and antiviral heterogeneity against either influenza viruses or PRRSV (7). For example, the typical porcine IFN-α/β subtypes, which generally has an activity at 10^3^-10^4^ U/μg/ml against PRRSV in porcine AMs and simian MARC-145 cells, had little activity against influenza viruses in human and mouse cells. The newly characterized porcine IFN-ω subtype comprises seven members, IFN-ω1 to IFN-ω7, who exert a broad antiviral spectrum in tests against influenza viruses and PRRSV in cells from mice, monkeys, pigs and humans. Porcine IFN-ω1 had a weak antiviral activity lower than most IFN-α/-β subtypes; IFN-ω5, in contrast, exerted a much higher antiviral activity (100–1,000-fold higher than IFN-α1) against PRRSV and influenza viruses in documented tests (7, 13). Studies thus indicate a rapidly evolving porcine IFN system with diversified subtype-specific antiviral activity, which warrants further investigations to optimize IFN-based antiviral design per the different virus-host interactions (7, 13).

Small RNAs (sRNAs) refer to a group of short (<200, and usually 18-30 nt in length) and non-coding RNA species. There are about seven families of sRNAs that mainly include microRNA (miRNA), small interfering RNA (siRNA), small nuclear RNA (SnRNA), small nucleolar RNA (snoRNA) and those derived from rRNA, tRNA, or fragments of transcribing genetic elements (14, 15). Among them miRNAs and siRNA have been mainly studied for regulatory roles participating in various cellular processes, including RNA splicing, modification, degradation, and translational arrest. Small RNAs, especially miRNA are known to modulate the cell response to virus infections (14, 15). Studies have provided evidence that miRNAs are pivotal in modulating macrophage polarization, differentiation, cytokine production, and inflammatory regulation (16). Over a dozen miRNA, including miR-21/-22/-146a/-155/-451 have been implicated in cellular processes of macrophages (16),which may link to AMs’ susceptibility and response to viral infections. The miRNA that play key roles in regulation of the AM response to IFNs and viral infection have not been critically investigated although several miRNA (such as miR-339-5p/-181d-5p) were differentially expressed in other cell types upon PRRSV infection (17–25). Similarly, a plethora of miRNA including miR-19/-122/-155 have been implicated in both IFN production and action signaling; however, no miRNA has been directly associated to differential antiviral regulation per different IFN subtypes and PRRSV strains with heterogeneous pathogenicity (17–28).

Here we aimed to characterize the small RNA composition using a whole transcriptome procedure (29). Differential miRNA responses were examined in porcine alveolar macrophages (PAMs) infected by two PRRSV strains (a live-attenuated vaccine strain and its relevant pathogenic PRRSV isolate) or treated with three IFN subtypes (IFN-α1/-ω1/-ω5, which have shown diverse antiviral activity) (3, 7). The data demonstrated relatively complete and comparable sRNA composition across the different treatments. Approximately 2% sRNA were differentially expressed in the antiviral treatments. Analyses on miRNA profiled 386 miRNA, including 331 known and 55 novel porcine miRNA sequences, of which most can be assigned to miRNA families conserved throughout mammalian species indicating cross-species validation (30). Functional analyses of significantly differentially expressed miRNA (SEM) across the different antiviral treatments demonstrated SEM expression patterns depend on the virus pathogenicity and antiviral potency of the tested IFN subtypes. We further articulated SEMs that were expressed commonly or uniquely in different treatments, and experimentally validated some SEMs including miR-10b and particularly miR-9-1 for their effect in in regulation of differential antiviral reactions. In summary, this study provides a general examination of porcine sRNA composition and pinpoints key SEMs underlying antiviral regulation in PAMs infected by a typical respiratory RNA virus. In turn, it evokes the potential of IFN-based antiviral and vaccine design for control of PRRS (3, 7, 12).

## Materials and methods

### Ethics statement and animal cells

No living animals were involved in this study; the porcine primary cells used were cryopreserved samples from previous studies (3, 7). The Institutional Biosafety and Institutional Animal Care and Use (IBC and IACUC) committees approved all recombinant DNA procedures and animal procedures. Porcine alveolar macrophages (PAMs) were collected using a lavage protocol from porcine lungs of 4- to 6-week-old pigs with 300 ml/each of 10 mM PBS (pH7.4) (3, 7). PAMs were isolated from the lavage fluid within 4 h after collection by centrifugation at 400×g for 15 min, and then isolated by plastic adherence (3, 7). Cells were used immediately or cryopreserved in Recovery™ cell culture freezing medium (Invitrogen, Carlsbad, CA) in liquid nitrogen until use. A monkey kidney cell line (MARC-145) used for PRRSV rescue and antiviral assays was purchased from ATCC and cultured following ATCC’s instructions or as previously described (3, 7).

### Virus infection, IFN treatment and sample preparation for transcriptomic analyses

PAMs were plated in RPMI medium with 10% FBS plus 1×penicillin/streptomycin and fungizone (Thermofisher) in 6-well culture plates. After being cultured overnight and floating dying cells removed, adherent PAMs in duplicated wells were: (1) mock-stimulated, (2) stimulated with porcine IFN-α1, IFN-ω1, and IFN-ω5 (Kingfisher, Saint Paul, MN) in a culture medium at 20 ng/ml for 5 h, or (3) infected with a wild-type PRRSV P129 strain (AF494042) or a relevant MLV vaccine strain (Ingelvac PRRS MLV vaccine, Boehringer Ingelheim Vetmedica) (12). Both PRRSV viral strains are classical PRRSV2 strains. The virus was infected at a multiplicity of infection (MOI) of 0.1 for 5 h, and washed twice with fresh culture medium prior to RNA and protein extraction (3, 31). BSA at 20 ng/ml in the culture medium was added to cultures of the mock-stimulated control.

### Small RNA transcriptomic analysis (sRNA-Seq)

Total RNA was extracted from ~2.5×10^7^ cells of each treatment using a column-based RNA/DNA/protein purification kit (Norgen Biotek, Ontario, Canada). RNA integrity and concentration were evaluated with a NanoDrop 8000 spectrometer (NanoDrop, Wilmington, DE) and an Agilent 2100 Bioanalyzer (Agilent Technologies, Santa Clara, CA) to ensure RNA samples with A260/A280>1.8 and RNA integrity number (RIN) >7.0 qualified for construction of sequencing libraries. Total RNA was fractionated into mRNA and sRNA fractions. A TruSeq Small RNA Sample Preparation Kit (Illumina, San Diego, CA, USA) was used for the library preparation following the manufacturer’s protocol. The libraries were sequenced using the Illumina HiSeq 2000 platform (Illumina, San Diego, CA, USA) for paired-end 50 bp at Novogene (Sacramento, CA). Finally, 22-28 Mb sequence was generated for each sample. The bioinformatics procedure for sRNA-Seq is illustrated in **Figure S1** and programs used are listed in **Table S1**. In brief, clean reads were obtained by filtering out a small portion (<1%) of adaptor contamination and low-quality reads, and length filtering to enrich the sRNA population (**Tables S2**, **S3**). The sRNA reads were then mapped to the swine reference genome through NCBI genome portal (http://www.ncbi.nlm.nih.gov/RefSeq/) (**Figure S2**), and classified based on the molecular/structural patterns of each type of sRNA species (**Figure S3**). Further analyses focusing on miRNA were performed regarding hairpin structures, family clustering, differential expression and target gene prediction. Differential expression of miRNA were cross-sample statistically analyzed and normalized by transcripts per million reads (TPM). The P-value corresponded to a differential gene expression test where FDR (False Discovery Rate) determined the threshold of the P-value in multiple tests. The functional classification of miRNA target genes was carried out through Gene Ontology and KEGG pathway analyses using the DAVID web tool (3, 29–31). The dataset was deposited in the NIH Short Read Archive linked to a BioProject with an accession number of PRJNA882823.

### Quantitative RT-PCR assays

Quantitative RT-PCR (qRT-PCR) assays were conducted as described (3, 7, 31). In brief, assays were performed in a 96-well microplate format using a QuantStudio™ 3 Real-Time PCR System (Thermofisher) with validated primers (**Supplemental Excel Sheet**). Reactions were performed with a SYBR Green RT-PCR kit (Qiagen, Valencia, CA) with 500 ng of total RNA in a 20-μl reaction mixture. Specific optic detection was set at 78°C for 15 s after each amplification cycle of 95°C for 15 s, 56–59°C for 30 s and 72°C for 40 s. Cycle threshold (Ct) values and melting curves were monitored and collected with the included software. Relative gene expression was first normalized against Ct values of the housekeeping gene (GAPDH), and compared with the expression levels of control samples (3, 7, 31).

### Target prediction and validation of selected miRNA on porcine gene targets in IFN signaling

The miRNA prediction and RNA structure prediction were analyzed using FindMiRNA and FoldRNA programs, respectively, through an online bioinformatic suite at http://www.softberry.com. The miRNA target prediction on the 3’-UTR of the various porcine genes were performed using three RNA analysis programs through an online BiBiServ Service (https://bibiserv.cebitec.uni-bielefeld.de/). The sequences of 3’-UTR regions and information about the transcripts of porcine genes/transcripts were extracted from the gene annotations at Reference genome/transcripts of Sscrofa11.1 version through NCBI genome ports (https://ftp.ncbi.nlm.nih.gov/genomes/all/GCF). The GenBank accession numbers of analyzed genes/transcripts are listed in i **Supplemental Table X**. The siRNA mimics and antisense inhibitors of several representative miRNA of SEM shared or unique among the samples, were synthesized and transformed into PAMs to evaluate the RNA interference effect against predicted corresponding porcine gene targets. The effect of the selected miRNA on PRRSV replication was determined by RT-PCR detection of ORF7 expression. The siRNA mimics and inhibitors were synthesized and transformed as previously described (32). In brief, the sense and antisense sequences of the siRNA were synthesized at IDT (Coralville, Iowa) together with an AlexaFluor-488 (AF488) labeled scramble siRNA, which was designed to serve as control siRNA and allow transfection optimization. PAM cells were cultured as described in a 24-well plate and transfected with Oligofectamine (Invitrogen to attain >90% transfected ratio as estimated by the AF488-scramble siRNA (30). Forty-eight hours after siRNA transfection, cells in different wells were collected for RNA extraction and gene specific RT-PCR was used to quantify the expression of target genes as described above. RNA samples used for RT-PCR assays were treated with RNase-free DNase I (NEB) to remove potential DNA contamination (3, 7, 32).

### Statistical analysis

Statistical analysis was completed using the SAS package (Company information). One-way analysis of variance (ANOVA) and Tukey’s *post hoc* test, as well as a two-sample F test was applied for significant evaluation between samples/treatments. A probability level of p<0.05 was considered significant (3, 7, 32).

## Results and discussion

### Comparable total and unique sRNA compositions across the PAM samples

Direct viral infection and passive interferon treatments represent major ways to induce the antiviral state in cells, particularly primary macrophages that serve as primary interferon producers in antiviral defense (5, 6). However, transcriptomic comparison has been seldom studied in the PRRSV-swine model. Based on our and others previous characterization (3, 7, 11, 13), the present study was designed to include two relevant PRRSV-2 strains with different pathogenicity and three interferon subtypes characterized with divergent antiviral activity to represent the swine interferon family. **Tables 1**, **2** provide general data about the qualified sRNA-Seq reads and sRNA composition across the analyzed PAM samples. For each sample, over 20M clean reads were generated to achieve genome-wide sRNA coverage and cross-sample comparability for potential differential expression. Of all treatments, PAMs infected by the pathogenic virus (P2-P129) generated the highest ratio of unique sRNA reads compared with the lowest one in PAMs treated with IFN-ω1 (P5), a weak antiviral IFN of porcine IFN-ω subtype as previously demonstrated (7, 13). The other four treatments were in the median of unique reads (**Table 1**).

**Table 1:** Type and quantity of sRNA reads and comparability among samples.

Sample*^1^	Total reads^2^	Total bases (bp)^3^	Uniq reads^4^	Uniq bases (bp)^5^
P1 (Ctrl)	21648455	501066839	405872 (1.9%)	9712569
P2 (P129)	24486844	588118302	**500458 (2.0%)**	13360663
P3 (MLV)	26139112	612027807	500630 (1.9%)	12376077
P4 (IFNα1)	22282175	520972685	406264 (1.8%)	10157086
P5 (IFNω1)	25470808	600884051	**388637 (1.5%)**	10125748
P6 (IFNω5)	27776163	647251618	494363 (1.8%)	12125389

(1) Sample: Sample ID/Treatment; (2) Total reads: Total number of sRNA reads; (3) Total bases (bp): Total reads multiplied by sequence length; (4) Unique reads: Types of sRNA; (5) Unique bases (bp): Unique reads multiplied by sequence length.

**Table 2:** Summary of small RNA (sRNA) annotation*.

Types	P1 (Ctrl)	P1(%)	P2 (P129)	P2(%)	P3 (MLV)	P3(%)	P4 (IFNα1)	P4(%)	P5 (IFNω1)	P5(%)	P6 (IFNω5)	P6(%)
Total^1^	19760012	100.00%	21676078	100.00%	23592435	100.00%	20078270	100.00%	22662197	100.00%	25001252	100.00%
known_miRNA^2^	17714665	**89.65%**	18230101	84.10%	20778435	88.07%	17953944	89.42%	20249333	89.35%	22127631	88.51%
rRNA^3^	409591	2.07%	569208	2.63%	531763	2.25%	378064	1.88%	455826	2.01%	667166	**2.67%**
tRNA^3^	6825	0.03%	11031	**0.05%**	9455	0.04%	7569	0.04%	8435	0.04%	9873	0.04%
snRNA^3^	14398	0.07%	17048	**0.08%**	17836	0.08%	13351	0.07%	14482	0.06%	20571	0.08%
snoRNA^3^	180276	0.91%	337426	**1.56%**	264358	1.12%	220300	1.10%	288043	1.27%	244501	0.98%
Repeat^4^	207041	1.05%	511065	**2.36%**	335728	1.42%	214678	1.07%	278178	1.23%	285854	1.14%
novel_miRNA^5^	12786	0.06%	8762	0.04%	14523	0.06%	15421	**0.08%**	11404	0.05%	20874	**0.08%**
exon:+^6^	420786	2.13%	824378	**3.80%**	606062	2.57%	504336	2.51%	593494	2.62%	599770	2.40%
exon:-^6^	256691	1.30%	332377	**1.53%**	346203	1.47%	252196	1.26%	216306	0.95%	352800	1.41%
intron:+^6^	233254	1.18%	387720	**1.79%**	322917	1.37%	228469	1.14%	252738	1.12%	313408	1.25%
intron:-^6^	84259	0.43%	152999	**0.71%**	109282	0.46%	88186	0.44%	91932	0.41%	102021	0.41%
Other^7^	219440	1.11%	293963	**1.36%**	255873	1.08%	201756	1.00%	202026	0.89%	256783	1.03%

All small RNAs are mapped.The sRNAs were transversed to ensure each annotation was unique. The order is: Known miRNA > rRNA > tRNA > snRNA > snoRNA > repeat > gene > novel miRNA. Items: (1) total: The quantity of sRNA reads mapped to genome. (2) known_miRNA: The number and percentage of sRNAs reads mapped to known miRNA. (3) rRNA/tRNA/snRNA/snoRNA: The number and percentage of sRNAs reads mapped to rRNA/tRNA/snRNA/snoRNA. (4) repeat: The number and percentage of sRNAs reads mapped to repeat regions. (5) novel_miRNA: The number and percentage of sRNAs reads mapped to novel miRNA. (6) exon: +/exon: -/exon: +/intron: -/intron: The number and percentage of sRNAs reads mapped to exon (+/-) and intron(+/-). (7) other: The number and percentage of sRNAs reads mapped to the genome but could not mapped to known miRNA, ncRNA, repeat, novel miRNA, exon/intron. Bold and underlined indicate the highest and lowest percentages, respectively, of each class of sRNA compared among the six treatments.

**Figure 1:**
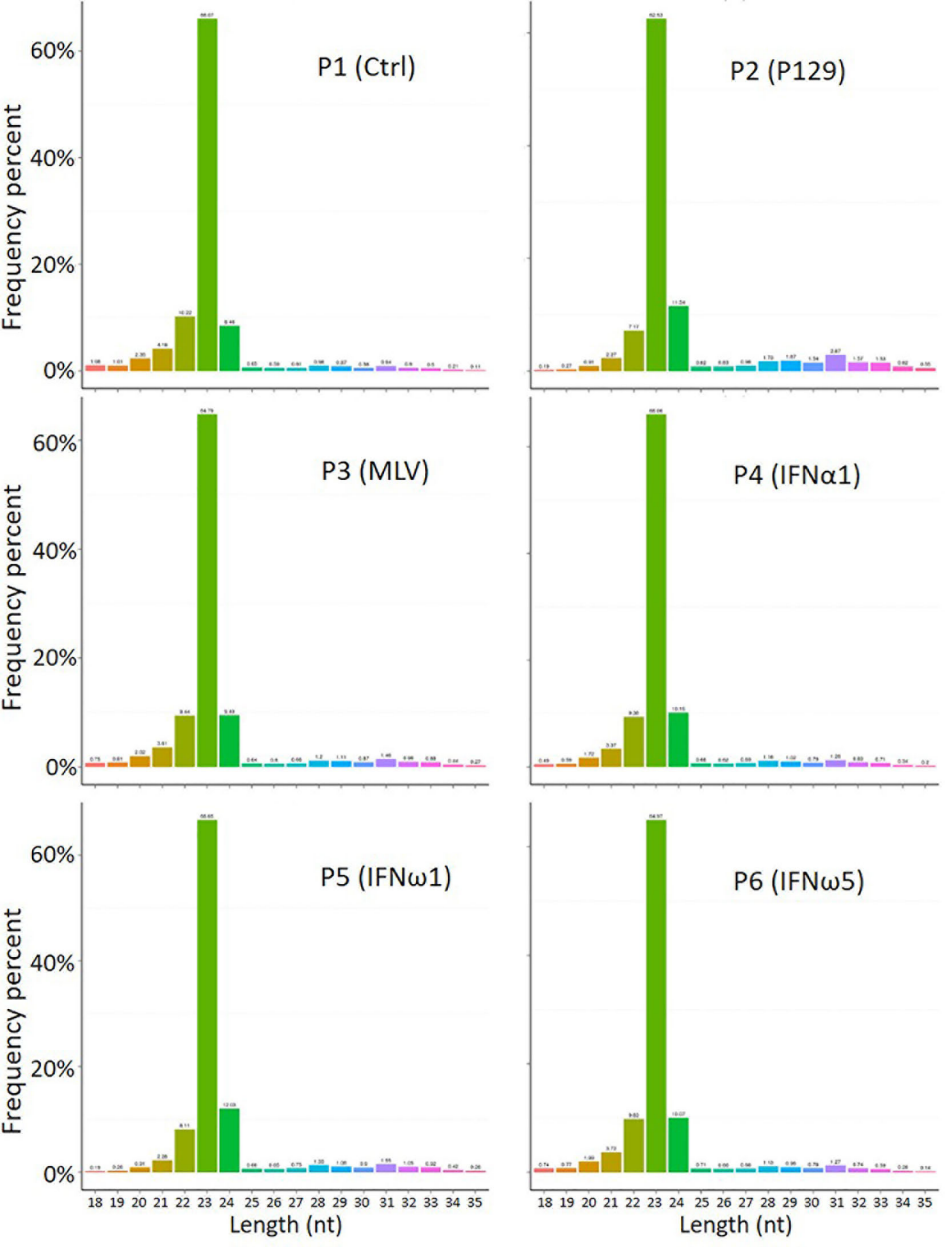
Length distribution of total sRNA profiled by the WTS. The abscissa is the length of sRNA reads, the ordinate is the percentage of one length read accounted for total sRNA. Shown is the majority (>80%) at 21-24 nt of typical miRNA, and comparable across the samples.

The profiled total sRNA showed a typical length distribution of sRNA. The majority (>80%) of the sRNA is at 21-24 nt of typical miRNA, and a highly comparable across the samples among the different treatments on PAMs (**Figure 1**). As were the sRNA reads distribution per chromosome as shown on the Circos diagrams (**Figure S2**). Randomly sampled 10,000 sRNA reads from each samples generated uniformly comparable distribution maps on the tested 10 longest contigs (or scaffolds) of the current swine reference genome, indicating a similar sRNA composition (**Figure S2**). In addition to profile typical regulatory sRNA classes including miRNA, rRNA, tRNA, snRNA, and snoRNA, total sRNA were also aligned to different repeat sequences and repetitive reads of sRNA were refined to prevent redundancy in counting accuracy (**Figure S3**). The collection of sRNA sequencing might include a trace amount of degraded fragments of mRNA. We also annotated the sRNA reads against both exon and intron regions of mRNA transcript database to define them as much as possible (**Table S5**). These sequences were removed to eliminate interference with new miRNA prediction, as these transcript regions contained much less miRNAs compared to the intergenic region, and therefore should be treated differently (29).

The overall classification and composition of sRNA profiled using the sRNA-Seq are presented in **Table 2**. Overall, the data showed that nearly 90% of sRNA were annotated as miRNA species, of which the majority were known miRNA in the miRbase (33).A minor portion (0.04-0.08%) of sRNA, with characteristic miRNA hairpin structure and not included in miRbase, were defined as novel porcine miRNA (33). In total, 331 known and 55 novel porcine miRNAs were profiled from the PAM samples, which provided 44% of all miRNA determined in various tissue/cell types of pigs (**Tables 3**, **4**). Cross-species comparison clustered most of these porcine miRNA into miRNA families that were conserved across different bilateral animals, especially vertebrates (**Table S6**) (30). The other classes of sRNA were about 10% with each class within the range of 0.03-2.7%, which had an enrichment order rRNA >snoRNA> snRNA > tRNA. The remaining sRNA included those mapped on repetitive elements (repeat) and exon or intron regions of transcript fragments (exon or intron) (**Table 2**). Cross-sample comparison of the percent of each class of sRNA also revealed that PAMs infected by the pathogenic virus (P2-P129) had the lowest ratio of miRNA verse the total sRNA (underlined; 84% *vs* ~89% in other treatments). In contrast, the PAMs of mock-treated control (P1) and weak antiviral IFN-ω1 (P5) sustained a near naïve status, which had the highest ratio (~89%; bold) of miRNA and lowest ratios (underlined) of other classes of sRNA. The other treatments, MLV infection or IFN-α1/-ω5 stimulation, had intermediate ratios of miRNA and other classes of sRNA as shown in **Table 2**. Using Venn diagrams, the common and specific reads of the total or sample-unique sRNA between samples were compared. Although the difference was diminished to some extent on the total sRNA scale, the P2-P129 infected sample had the lowest ratios of common and unique sRNA compared with the control (P1); and other treatments, including the IFN-ω1 treatment (P5) had a higher ratio of common/unique sRNA as to the control (P1) (**Figure S4**). Collectively, present sRNA-Seq generated a reliable and comparable sRNA composition across the PAM samples; and differential analysis clearly indicated an aberrant status relevant to miRNA-suppression verses upregulation of other sRNA classes in the sRNA turnover mediated by pathogenic PRRSV infection in PAMs. The contribution of this distortion on sRNA biogenesis and degradation by PRRSV infection warrants further investigation (21, 28).

**Table 3:** Summary of known sRNA mapped in each sample.

Types*	Total	P1 (Ctrl)	P2 (P129)	P3 (MLV)	P4 (IFNα1)	P5 (IFNω1)	P6 (IFNω5)
Mapped mature^1^	331	304	304	**314**	304	289	307
Mapped hairpin^2^	304	284	277	**289**	284	272	283
Mapped uniq sRNA^3^	18504	3189	2883	**3300**	3082	2751	3299
Mapped total sRNA^4^	117054109	17714665	18230101	20778435	17953944	20249333	**22127631**

(1) Mapped mature: The number of sRNAs align to miRNA mature sequence; (2) Mapped hairpin: The number of sRNAs align to miRNA hairpin sequence; (3) Mapped uniq sRNA: The number of mapped unique sRNAs; (4) Mapped total sRNA: The number of mapped total sRNAs. Refer to **Figure S3** for a diagram of a secondary structure/mature sequence of typical miRNA. The highest and lowest numbers among the samples for each class of sRNA were shown bold or underlined, respectively.

**Table 4:** Summary of novel sRNA mapped in each sample*.

Types	Total	P1 (Ctrl)	P2 (P129)	P3 (MLV)	P4 (IFNα1)	P5 (IFNω1)	P6 (IFNω5)
Mapped mature	55	**45**	35	42	44	37	44
Mapped star	17	6	8	10	10	5	**11**
Mapped hairpin	55	**46**	37	43	**46**	38	**46**
Mapped uniq sRNA	714	124	100	130	122	100	**138**
Mapped total sRNA	83770	12786	8762	14523	15421	11404	**20874**

Novel miRNA prediction. The characteristic hairpin structure of miRNA precursors can be used to predict novel miRNA, conducted by miREvo (Wen et al., 2012) and mirdeep2 (Friedlander et al., 2011). The highest and lowest numbers among the samples for each class of sRNA were shown bold or underlined, respectively.

### Known and novel miRNA/sRNA profiled in PAMs

**Tables 3**, **4** summarize the numbers of all known and novel sRNA, especially miRNA, which were mapped in each sample. For the known sRNA listed in **Table 3**, about 90% reads of >20M sRNA reads were aligned to the mature (or hairpin precursor) sequences of near 300 miRNA identified in pigs, with most miRNA only having one to a few copies and some top-enriched miRNA having over 100,000 copies detected by sRNA-Seq (**Table 5**). Collective data indicated that the sRNA-Seq profiled both rare and rich sRNA transcripts that were commonly or differentially expressed across the PAM samples. Of the known sRNA/miRNA mapped, the PAMs infected by the MLV strain (P3) expressed slightly higher numbers of known sRNA/miRNA, whereas the P5 of IFN-ω1-treated PAMs still contained the lowest numbers of known sRNA/miRNA (**Table 3**). In contrast, slightly more novel sRNA/miRNA were detected in the P6 of IFN-ω5 treatment (**Table 4**). More extensive or sRNA/miRNA-specific studies will be needed to test whether this general sRNA differential expression is associated with the respective samples across different treatments (21–29).

**Table 5:** Top enriched miRNA that each had >10,000 reads as exemplified from the control P1 sample.

Mature miRNA sequence detected(5'- to -3')*	Read numbers inControl PAMs	miRbase assignation	Note
UGAGAACUGAAUUCCAUg/aGGu/cOG	315476	ssc-miR-146a-5p	Site mutations
UGAGAACUGAAUUCCAUg/aGGu/cOGU	115713	ssc-miR-146a-5p	Site mutations/new isoform
UUCACAGOGGCUAAGOUCCG	147667	ssc-miR-27a	
UAGCOUAOCAGACUGAUGUUGA	368785	ssc-miR-21-5p	
UAGCUUAUCAGACUGAUGOUGAc	13262525	ssc-miR-21-5p	1nt ext isoform
UAGCUUAUCAGACUGAUGOUGAca	679529	ssc-miR-21-5p	2 nt extisoform 1
UAGCUUAUCAGACUGAUGUUGAcu	480859	ssc-miR-21-5p	2 nt extisoform 2
UAGCUUAUCAGACUGAUGUUG	357848	ssc-miR-21-5p	1nt short isoform 3
UGAGGUAGUAGUUUGOGCUGUU	313245	ssc-let-7i-5p	
UGAGGUAGUAGAOUGOAOAGOU	297453	sse-let-7f-5p	
UGUAAACAUCCCCGACUGGAAGCU	140712	ssc-miR-30d	
ACUGGACUUGGAGUCAGAAGGC	112383	ssc-miR-378	
UUCAAGUAAUCCAGGAUAGGCU	239999	ssc-miR-26a	

*The nucleotide residues in lowercases, like “g/a” indicate the residue (g) in the consensus miRNA mutated to “a” in this detection, and those at the 3’- of several miR-21-5p isoforms indicate the difference from the consensus in miRBase database as noted.

This study identified over 50 novel porcine miRNA based on their characteristic hairpin structure of miRNA precursors. These novel miRNA were predicted using both their mature and hairpin precursor sequences, using the two recent miRNA-predicting programs of miREvo and mirdeep2 (34, 35). The novel porcine miRNA had no consensus entries in miRbase or other miRNA databases (33), which should be included after further validation at both molecular and function aspects (**Table 4**). In addition, this *de novo* sRNA-Seq also revealed site-mutations and isoforms of porcine miRNA as illustrated in **Table 5** for enriched miRNA in this study. For example, the two ssc-miR-146a-5p isoforms with 23 or 24 nt mature sequences, were nearly 3-fold differentially expressed from the control PAMs, and contain two site-mutations compared with the consensus sequence of ssc-miR-146a-5p at miRbase (33). More extensively, the ssc-miR-21-5p had six isoforms that were detected that differed in three nt at the 3’-terminus and again showed a broad range of differential expression in the PAMs. These results imply some understudied molecular complexity of porcine miRNA regarding individual polymorphism and potential functional divergence (36, 37).

### Correlation and differential analyses of miRNA expression across antiviral treatments

**Figure 2:**
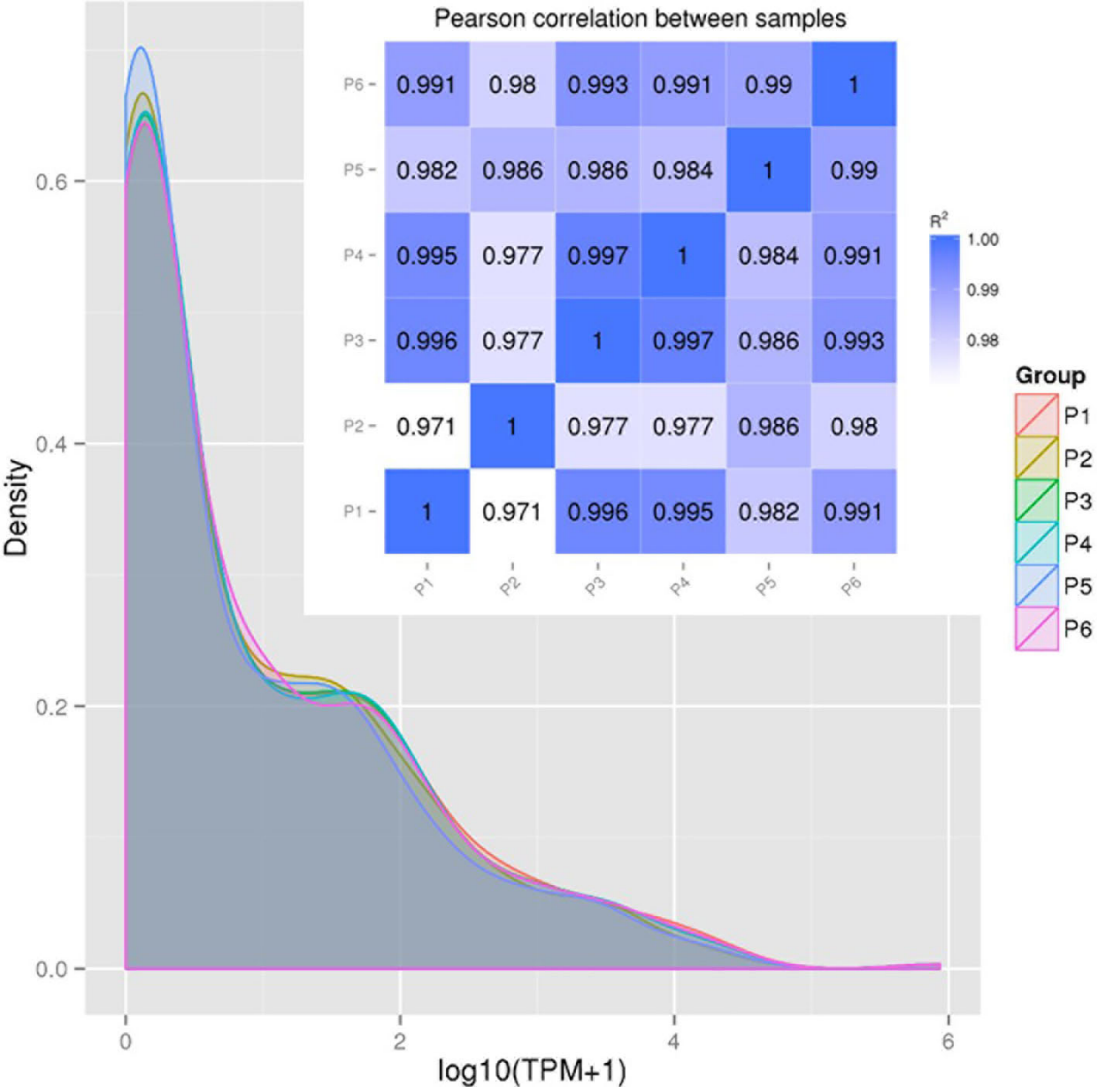
Correlation of miRNA expression levels between samples to show experimental repeatability for analysis of differential gene expression. miRNA expressions in each sample were statistically analyzed and normalized by transcripts per million reads (TPM). Shown are TPM density distribution patterns (shaded line chart) and Pearson Correlation (inset Pearson RSQ table) between samples. The closer the correlation coefficient is to 1, the higher the similarity between the samples. Encode suggests that the square of the Pearson correlation coefficient (R^2^ or RSQ) should be larger than 0.92 under ideal experimental conditions.

**Figure 2** provides the correlation analysis of miRNA expression levels between samples to show the experimental repeatability of differential gene expression. First, miRNA expressions in each sample were statistically analyzed and normalized by transcripts per million reads (TPM), independent on the length of sRNA. The TPM density distribution patterns in **Figure 2** illustrate that the six PAM samples shared most regions of the distribution pattern indicating that PAMs of all treatments had most miRNA maintained the basic cellular expression in PAMs of all treatments. Specifically, miRNA that had the log_10_(TPM+1) values within the low (<0.4), mid (0.8-3.2) or high (>4.8) regions showed higher divergence indicating differential expression among the six antiviral treatments in PAMs. For example, the P5-IFNω1 treatment had the highest density of rarely expressed miRNA [log_10_(TPM+1)<0.4), followed by the P2-P129, P3-MLV, P4-IFNα1, and P6-IFNω5 treatments in a decreasing order. In contrast the P6-IFNω5-treated PAMs were stimulated to have more miRNA with the highest log_10_(TPM+1)>4.8.

The Pearson correlation coefficient (R2 or RSQ) is shown in the inset Pearson table in **Figure 2** and **Figure S7**. Most of our inter-sample RSQ for miRNA expression were larger than 0.97, indicating a critical experiment control across the samples, and shared expression patterns of the miRNA that correlates between the samples. Hence, the minor portion of sample- and miRNA-specific differential expression, which were hypothesized to underlie the observed difference of cellular responses across the samples, were pinpointed for further examination (20–29).

**Figure 3:**
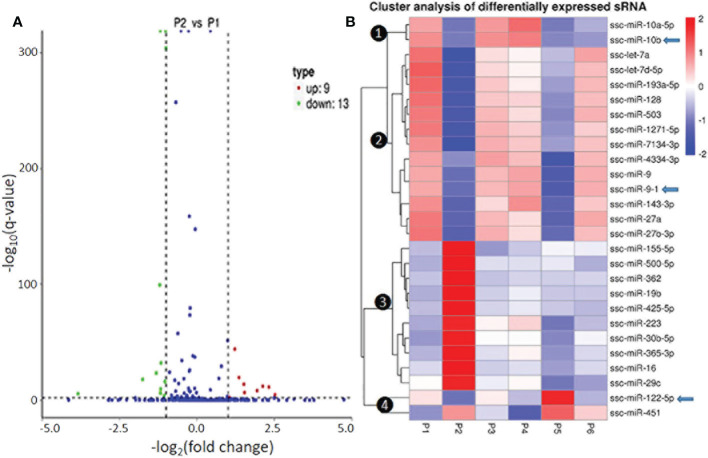
Differential expression and cluster analysis of differentially expressed miRNA (SEM) in different samples. **(A)** Volcano Plot to show DEMs in P2 (MLV) compared with P1 (Control). The other pairs of comparison are listed in **Figure S7**. The X-axis shows the fold change in miRNA expression between the different samples, and the Y-axis shows the statistical significance of the difference. Statistically significant differences (|log2(FoldChange)| > 1 and q-value < 0.01) are represented by red or green dots for up- or down-regulated miRNA, respectively. **(B)** Hierarchical clustering of significantly DEMs (exemplified of 157 families in total, see **Supplemental Data Sheet** for detail) in compared samples. miRNAs within the same cluster have the same trend in expression levels under different conditions. In addition to the TPM cluster, K-means and SOM were also used to cluster the log2(ratios) (data not shown). Arrows indicate miRNA that show differential expression and were selected for functional studies.

In **Figure 3** and **Figure S8**, we present the differential expression and cluster analysis of significantly differentially expressed miRNA (SEM) in different samples. The statistic defaults for the SEM were defined as |log_2_(Fold Change)| > 1 and q-value < 0.01), and represented by red or green/blue for up- or down-regulated miRNA, respectively, in Volcano plots and heatmap, (**Figure 3** and **Figure S8**). Notably, if qualified by only one parameter of either log2(Fold Change)| > 1 or q-value < 0.01, many more SEM (157 miRNA families in total; see **Supplemental Data Sheet**) were included for differential comparison but could cause noise for scoping the true SEM (29). In general, the P2-P129 infection and P5-IFNω1 treatment regulated more SEMs compared with other samples. Hierarchical clustering of true SEM in compared samples using heatmap analysis was shown in **Figure 3B**, where differently colored patterns represented different SEM clusters, and miRNAs within the same cluster had a similar differentiating trend in expression levels under differing conditions. Across the samples, data showed that SEM shared similar up- or down-regulated expression between the P1 control and each of the P3-MLV infection, P4-IFNα1 or P6-IFNω5 treatments, except ssc-miR-10a/b and ssc-miR451 with a reversed regulation compared to that of P1 control. In contrast, the up- or down-regulation patterns of almost all SEM shared by the three treatments above was almost completely reversed to down- or up-regulation as seen in the P2-P129 infected PAMs. In the P5-IFNω1-treated PAMs, only the up-regulated SEMs became mostly down-regulated, and most down-regulated ones stayed. Findings implicate that PAMs, which were treated using a weak antiviral IFNω1 (P5) and infected by PRRSV (P2), showed a higher differential expression of miRNA than the MLV infection or IFNα1/ω5 that were stronger antivirals (7). The reversed patterns of the up- or down-regulated SEMs by these two relevant PRRSV strains in macrophages hypothetically correlate to the clear divergence of the viral pathogenicity and in antiviral stimulation, and constitute a transcriptomic signature of non-coding RNA response underlying the virus-host interaction subjected by PRRSV. In this regard, the three porcine IFN subtypes tested represent the broad spectrum of antiviral activity of porcine IFN family against PRRSV in tested cells: IFN-ω1/-ω5 represent IFN subtypes with the weakest and highest anti-PRRSV activity, respectively; and IFN-α1 being a typical type I IFN subtype exert a mode antiviral protection as characterized (7, 13). Hence, our observation was the wild-type strain and IFN-ω1 show almost the same phenotype, while similar SEM patterns were found in the treatments by the vaccine strain and antiviral IFN-α1/-ω5 subtypes, indicating a similar antiviral regulation in macrophages between comparable IFN subtypes and/or viral strains. This further implicates an important role of miRNA response in IFN- and MLV-mediated antiviral responses at least in macrophages. As the dramatic effect of the pathogenic virus infection on miRNA expression was expected, the surprising observation in IFNω1-treated PAMs may correlate to the broad effect of IFN-ω subtype beyond the antiviral activity, such as those in cell growth and metabolic regulation (38, 39). However, porcine miRNA previously associated with PRRSV infections, including ssc-miR-23, ssc-miR-24-3p, ssc-miR-30c, ssc-miR-181, ssc-miR-210, and ssc-miR-331-3p, were not among the list of the SEM profiled in this study (18–28). This perhaps comes from the discrepancy of the cell type/status, virus pathogenicity, treatment conditions, or the defaulted setting of programs used for data processing (21, 28, 29).

Of the four clusters of SEM labeled on the phylogenic tree of the heatmap (**Figure 3B**), Cluster 3 contained 10 miRNA phenomenally upregulated in the P2-P129 infected PAMs but generally suppressed in PAMs subjected to other conditions, indicating that these 10 miRNA could be involved in PRRSV-PAM interaction and underlying PRRSV pathogenesis (18–28). Twelve miRNA were clustered in Cluster 2, which were generally or significantly suppressed in the PAMs treated by the weak antiviral IFN-ω1 (P5) and infected by the pathogenic PRRSV (P2). We observed that this group of miRNA mirrored the miRNA that played a vital role in macrophage activation and IFN signaling in previous studies (16, 17). This indicates their potential role in polarization of macrophages and regulation of an antiviral state corresponding to different IFN stimuli. Cluster 1 and 4, each consisted of only two miRNA, showed differential expression across the six treatments. The miR-10a-5p/-10b of Cluster 1 and miR-451 of Cluster 4, were contrastingly regulated by the IFN-α (P4) and IFN-ω subtype (P5 and P6). In addition, the miR122-5p in the Cluster 4 was distinguished by a contrasting expression pattern between P5 (IFN-ω1-treated) and P4/P6 treatments using two stronger antiviral IFNs of different subtypes (7, 13). Notably, a majority of these SEMs identified by the sRNA-Seq had predicted target sites on the consensus sequence of type 2 PRRSV (PRRSV-2) genome or the 3’-untranslated regions (UTR) of tested porcine gene transcripts, which include IFNAR1/2, JAK1, Tyk2, STAT1/2 and IRFs that are key in IFN signaling (**Table 6**) (21, 40). In addition, about half of these SEM have been well implicated in regulation of macrophage activation and IFN signaling by previous studies in humans or animals (16, 17).

**Table 6:** Top ranked SEM shared or unique in compared groups.

	Compared pairs (Log_2_ Fold change)					Target sites on PV-2 and pig ISGs*	Implicated in AM- or IFN-biology**
Common SE miRNA	P4 vs P1	P4 vs P2	P4 vs P3	P4 vs P5	P4 vs P6		
ssc-miR-9-1		1.3516	-1.3728			PV(2), IFNARl/2(1), IRFl/8(1)	IFN(STAT3),AM(TLR)
ssc-miR-10b		4.2402	-3.7285		3.8035	None	
**Unique SE miRNA**							
ssc-miR-7134-3p		1.0591				PV(l)	
ssc-miR-155-5p		-1.0555				PV(1),IFNAR1(2)	IFN(SCOS1),AM(TLR)
ssc-miR-425-5p		-1.083				PV(3),1FNAR1(2), IRF4/9(1)	
ssc-miR-16		-1.3603				STAT2(1)	
ssc-miR-500-5p		-1.9106				PV(2),STAT2(1),1RF9(1)	
ssc-miR-19b		-1.6516				PV(2)	IFN(TLR2,SCOS1)
ssc-miR-503		1.2805				STAT2(1)	
ssc-miR-362		-1.3365				TYK2(1). STAT2(2),I RF8(1)	
ssc-miR-30b-5p		-1.3328				IFNAR2(1),IRF6(1)	IFN(+)
ssc-miR-143-3p		1.1999				PV(1)	IFN(+)
ssc-miR-365-3p		-1.7771				PV(l ),IFNAR1(2), TYK2(1)	
ssc-miR-451		-3.1981				None	AM(M1-activation)
ssc-miR-10a-5p		1.5497				None	
**Common SE miRNA**	P5 vs P1	P5 vs P2	P5 vs P3	P5 vs P4	P5 vs P6		
ssc-miR-9-1	-1.2831		-1.2494	-1.3728	-1.3477	PV(2), IFNAR1/2(1).1RF1/8(1)	IFN(STAT3),AM(TLR)
ssc-miR-10b	-3.4099		-3.501	-3.7285		None	
ssc-miR-451	3.1812		2.3613	3.9652		None	AM(M1-activation)
ssc-miR-122-5p		2.7101		2.4297	2.4344	None	IFN(-)
**Unique SE miRNA**							
ssc-miR-503	-1.0699					STAT2(1)	
ssc-miR-16		-1.1457				STAT2(1)1	
ssc-miR-19b		-1.4051				PV(2)	IFN(TLR2,SCOS1)
ssc-miR-30b-5p		-1.4289				IFNAR2(1),IRF6(1)	IFN(+)
ssc-miR-500-5p		-1.3863				PV(2). STAT2(1), IRF(1)	
ssc-miR-365-3p		-2.135				PV(l), IFNAR1(2), TYK2(1)	
ssc-miR-29c		-1.6181				IFNAR2(1)	IFN(-)
ssc-miR-4334-3p			-1.0555			PV(3), STAT2(1),IRF4/5(1)	
ssc-miR-10d-5p				-1.2676		None	
**Common SE miRNA**	P6 vs P1	P6 vs P2	P6 vs P3	P6 vs P4	P6 vs P5		
ssc-miR-9-1		1.1828				PV(2). I FNAR1/2(1), IRF1/8(1)	IFN(STAT3),AM(TLR)
ssc-mi R-10b	-3.4273		-3.5448	-3.8035		None	
**Unique SE miRNA**							
ssc-mi R-155-5p		-1.0401				PV(1),IFNAR1(2)	IFN(SCOS1),AM(TLR)
ssc-miR-425-5p		1.3168				PV(3). IFNAR1(2). IRF4/9 (1)	
ssc-miR-16		-1.4683				STAT2(1)	
ssc-miR-500-5p		-2.2612				PV(2),STAT2(1),1RF9(1)	
ssc-miR-19b		-1.9508				PV(2)	lFN(TLR2,SCOS1)
ssc-miR-503		1.4366				STAT2(1)	
ssc-miR-362		-1.4287				PV(l). TYK2(1).STAT2(2), IRFB(l)	
ssc-miR-30b-5p		-1.4631				IFNAR2(1),IRF6(1)	IFN(+)
ssc-miR-36S-3p		-2.2045				PV(l). IFNAR1(2).TYK2(1)	
ssc-mi R-122-5p					-2.4344	None	IFN(-)
							

*The potential target sites on the viral genome was predicated using a typical PRRSV-2 genome (PV-2, NVSL-97-7895, NCBI Acc# AY54598.1). The 3-UTR of porcine IFNAR1, IFNAR2, JAK1, Tyk2, STAT1/2, and IRFs genes that are key in type I IFN signaling were tested using RNAhybrid. **The involvement in AM or IFN signaling was based on two references (16, 17), and up-/down-regulation (+/-) or a major signaling component targeted is indicated in the parentheses.

### Validation of transcriptomic expression using qRT-PCR assays for representative miRNA

**Figure 4:**
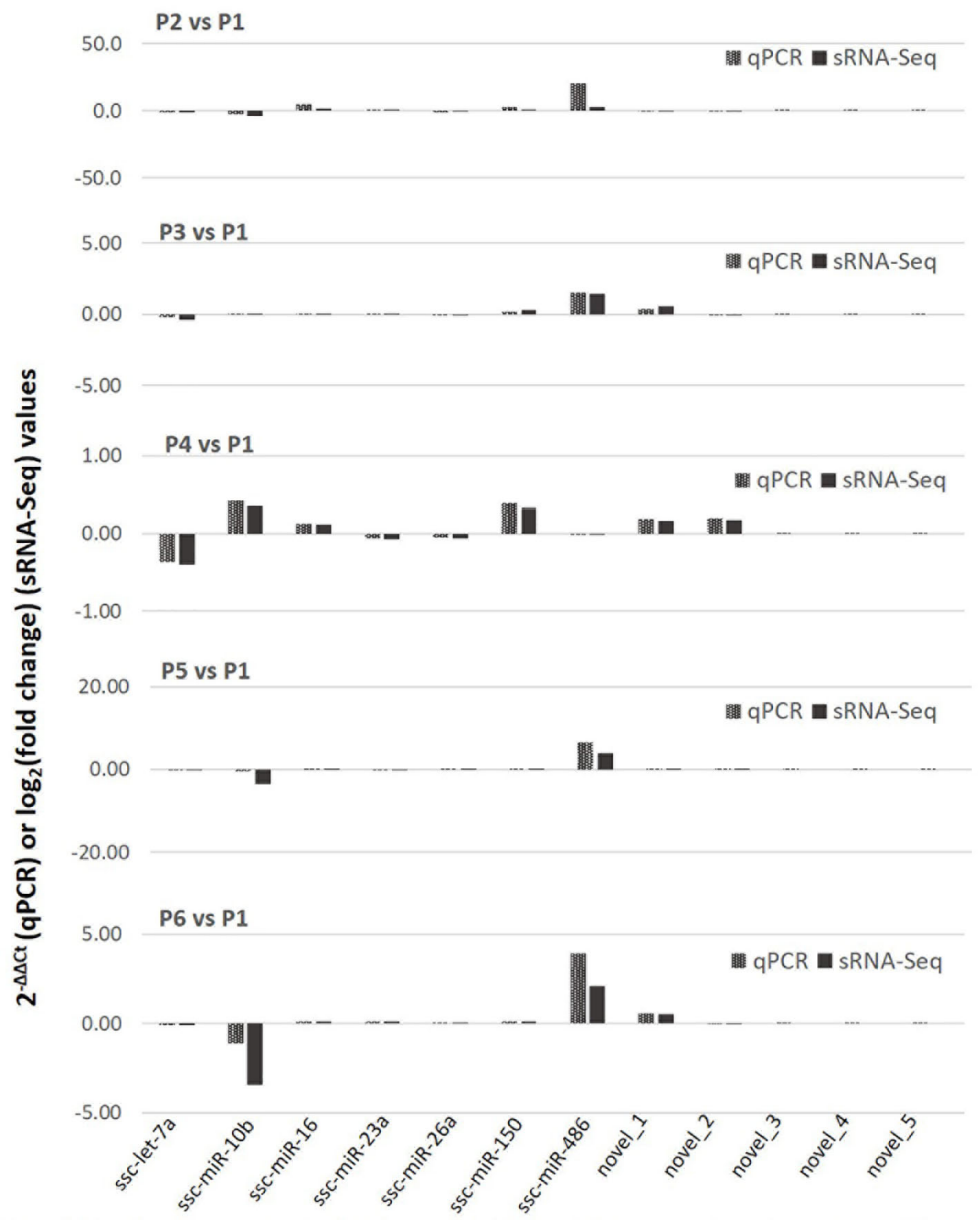
Differential expression of selected known and novel miRNA by sRNA-Seq was verified with a targeting quantitative RT-PCR (qPCR) assay. The y-axis scale indicates 2^−ΔΔCt^ (qPCR) or Log2(fold change) values relative to the control P1 sample. Aliquots of the cell RNA used for sRNA-Seq were analyzed using a SYBR green-based real-time RT-PCR assay. Ct values of the genes were normalized against Ct values of a housekeeping gene (coding for glyceraldehyde-3-phosphate dehydrogenase [GAPDH]) amplified from the same RNA samples to obtain 2^−ΔCt^ and 2^−ΔΔCt^ relative to the control. Data shows a general consistence of the differential expression of both known and novel miRNA revealed by the sRNA-Seq.

We further validated the differential expression using a quantitative RT-PCR (qRT-PCR) assay of selected known and novel miRNA, which represented both high and low expression levels profiled with sRNA-Seq (**Figure 4**). Data show a general consistency of differential expression in both known and novel miRNA with the sRNA-Seq, even though there was some quantitative discrepancy, which was acceptable given the relative quantitation in PCR compared with read-counting in sRNA-Seq. We also confirmed that despite about 55 novel porcine miRNA being revealed using *de novo* sRNA-Seq, most of them were expressed at a low level in the PAM samples and few of them were among the SEM cluster that showed significant stimulation (**Figure 4**). This may explain why these novel porcine miRNAs have not been well-documented in previously reports (40–46), and imply that they are probably involved in regulation of some uncommon cellular pathways (16, 17).

### Gene ontology analysis of miRNA-targeted genes

**Figure 5:**
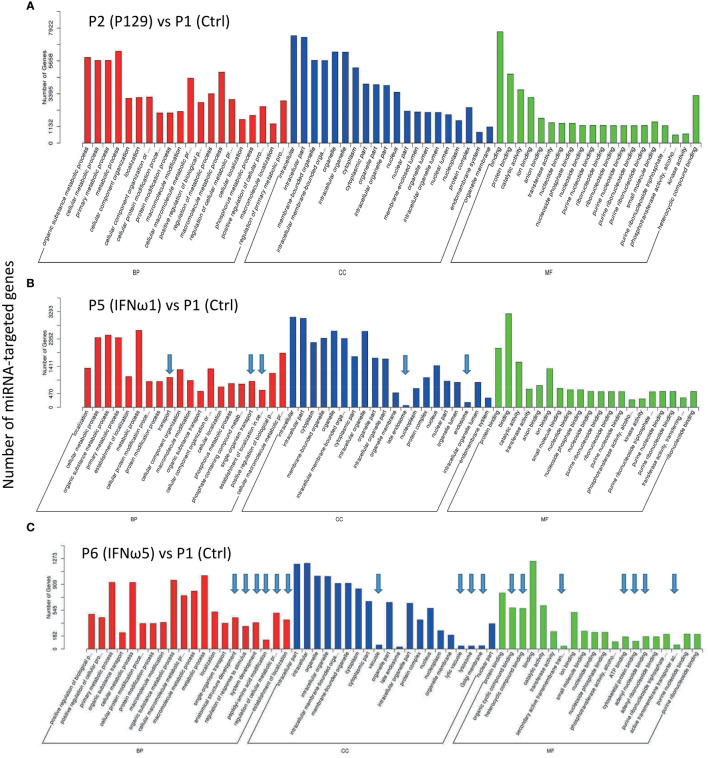
Gene Ontology (GO) analysis to unify miRNA-targeted genes in three main branches: cellular compartment (CC), molecular function (MF) and biological process (BP). Shown are GO terms with *p*-adj < 0.05 of significant enrichment in selected comparison of treated samples of P2 **(A)**, P5 **(B)**, and P6 **(C)** with the P1 control, and other samples’ comparison is provided in **Figure 9S**. Wallenius non-central hyper-geometric distribution was used to discover significantly enriched GO terms in target gene candidates relative to the reference gene background (http://www.geneontology.org/). Arrows indicate unique top GO items detected compared with the P2 *vs* P1.

SEM target genes were predicted and Gene Ontology (GO) and pathway enrichment analyses were performed. Each miRNA may target through perfect or “promiscuous” pairing with their seed region (position 2-7), hence, many mRNA targets can be regulated by one miRNA or different miRNA may regulate one mRNA at different sites (47). For example in humans, given approximately 2,500 human mature miRNAs and 22,500 human protein coding genes, 50 million potential pair-wise interactions between miRNAs and genes are predicted to be possible (48). Gene Ontology (GO) analysis unifies miRNA-targeted genes in three main branches: biological process (BP), cellular compartment (CC), and molecular function (MF). **Figure 5** shows top GO terms of significant enrichment (p-adj < 0.05) of the P129-infected sample (**Figure 5A**) or the two treated using IFN-ω1/-ω5 (**Figures 5B, C**) compared with the P1 control, and other samples’ comparison is provided in **Figure 9S**. In general, most GO items enriched by the SEM-targeted transcripts were concentrated in metabolic process and biomolecule localization/modification (BP), intracellular cytoplasm/organelle/nucleus (CC) and intermolecular binding (MF). Further determination revealed the unique GO items of BP, CC and MF groups included pathways: transport-/endosome-related in the two IFN-ω treatments, as well as several other pathways including anatomical structure development, regulation of response to stimulus, peptidyl-amino acid modification, ATP-binding, cytoskeletal protein binding being specifically in the IFN-ω5 treatment only (**Figures 5B, C**, arrows). In summary, the GO analysis of the SEM-targeted mRNA indicated most early effects of the antiviral regulation in PAMs, which include those underlying metabolic alteration, intracellular molecular relocation or interaction. Remarkably, the P2-infected and IFN-ω-treated (P5/P6) PAMs had many more SEM than the samples infected by a MLV (P3) or treated using the IFN-α subtype. Further studies are needed to determine whether the GO pathways specifically enriched in the virus-infected and IFN-ω-treated PAMs are responsible for the virus pathogenesis or the antiviral diversity induced by the unconventional IFN-ω subtypes (4, 7, 16, 17, 49).

### Pathway enrichment analysis of miRNA-targeted genes

**Figure 6:**
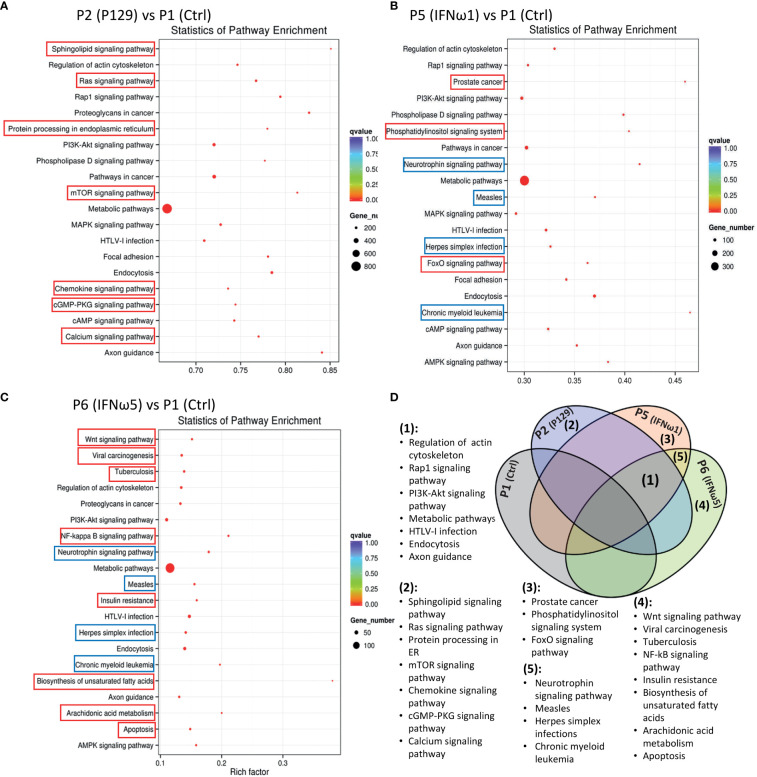
Scatter plots of pathway enrichment analysis of genes targeted by significantly differentially expressed miRNAs between the control P1 and the P129-infected **(A)** or treated with IFN-ω1 **(B)** or IFN-ω5 **(C)**. Pathway enrichment analysis was conducted based on the Kyoto Encyclopedia of Genes and Genomes (KEGG) database. The Y-Axis lists significantly enriched pathways and X-Axis shows the enrichment factor. Dot size represents the number of miRNA-targeted genes and the color indicates the scale of the q-value. Pathways framed indicated unique pathways enriched in each compared pair only (Red frame) or some compared pairs (Blue frame). **(D)** Venn diagram to summarize pathways commonly (1) unframed pathways in **(A–C)** or uniquely (2-5). Framed pathways in **(A–C)** enriched in the different comparisons.

After the general GO classification, we performed pathway enrichment analysis of genes targeted by SEM between the PAMs of different treatments (**Figure 6** and **Figure 10S**). The scatter plots of **Figure 6** and **Figure 10S** illustrate the top-ranked pathways enriched by the SEM-targeted genes. First, about two-third of the enriched pathways were common in the PAMs of different treatments, indicating basic PAM identity across different treatments (unframed pathways in each scatter plot and highlighted as Fraction 1 in the Venn diagram of **Figure 6D**). These common pathways included regulation of actin cytoskeleton, Rap1 signaling, PI3K-Akt signaling, metabolic process, HTLV-I infection, endocytosis and axon guidance, which were well implicated in cell physiology including cell adhesion, polarity, growth, survival, and differentiation in response to extracellular signals upon viral infection and cytokine stimuli (4, 16, 17, 48). Unique pathways were also revealed comparatively. For example, the unique pathways in the virus-infected P2 sample included sphingolipid signaling pathway, Ras signaling pathway, protein processing in endoplasmic reticulum, mTOR signaling pathway, chemokine signaling pathway, cGMP-PKG signaling pathway, and calcium signaling pathway. All these pathways have been implicated in immune regulation, antigen presentation, and inflammatory regulation (4, 16, 17, 48, 49). Compared with the PAMs infected by the MLV strain, which showed less SEM response, it is rational to suggest that PRRSV utilizes the cellular miRNA system to facilitate its infection and immunosuppression in PAMs (40). For the PAMs treated with IFN-ω1, three unique pathways were shown including prostate cancer, the phosphatidylinositol signaling system, and the FoxO signaling pathway, which fundamentally mediates cell proliferation, survival, and metabolism, as well as regulates cell migration, endocytosis, apoptosis, and oxidative stress resistance that critically underlies various immune responses. Previous studies showed a broad and superior antiviral activity of porcine IFN-ω5 (7). The pathway analysis of the IFN-ω5-treated PAMs revealed eight unique pathways, which were the Wnt signaling, viral carcinogenesis, Tuberculosis, NF-κB signaling, insulin resistance, biosynthesis of unsaturated fatty acids, arachidonic acid metabolism and apoptosis pathways (Framed with red line in **Figure 6C** and Fraction 4 in **Figure 6D**). All these IFN-ω5-specific pathways are associated with not only the immune responses pertinent to antimicrobial and inflammatory regulation (such as viral carcinogenesis, Tuberculosis, NF-κB signaling, biosynthesis of unsaturated fatty acids, and arachidonic acid metabolism), but also the physiological process involving cell growth, carcinogenesis and cell death. This distinguishes a multifunctional property of the understudied IFN-ω subtype emerging in mammalians and especially expanded in bats and ungulates (7). In this context, four KEGG pathways were unique and shared in PAMs stimulated by the two IFN-ω1/ω5 (Framed with blue line in **Figures 6B, C**, and Fraction 5 in **Figure 6D**). These IFN-ω-specific pathways included neurotrophin signaling, measles, Herpes simplex infections, and chronic myeloid leukemia, which again were concentrated in viral infection and myeloid cell differentiation but also the neurotrophin signaling at the interface of neuronal and immune regulation (4, 16, 17, 49). Previously, we have proposed to incorporate the IFN-induced antiviral state into the paradigm of macrophage activation statuses (3), which are traditionally classified as the classical M1 and alternative M2 statuses based on distinct cellular surface markers and secreted cytokines corresponding to different macrophage’s properties regarding phagocytic, inflammatory and antimicrobial capacity (3, 4). Recent studies,using extensive omics profiling, further classified the four activation statuses of macrophages, which include Status P1 to P4 to reflect macrophages prone to phagocytic, oxidative, inflammatory and remodeling activity, respectively (4). Still, it is hard for us to distinctly ascribe the PRRSV-infected or IFN-stimulated PAMs to a certain activation status, but they probably represent a mingled status among the Status P1 to P4 (4). Most studies about macrophage activation status were done using models primarily of bacterial infections or cytokine stimulation *in vitro*; therefore, further work is necessary to delineate the antiviral state in the scenario of activation status of macrophages, and this is imperative for dealing with bacterial/viral co-infections commonly confronted by tissue macrophages including lung macrophages (3, 4, 16).

### Functional articulation and validation of SEMs in antiviral regulation

**Figure 7:**
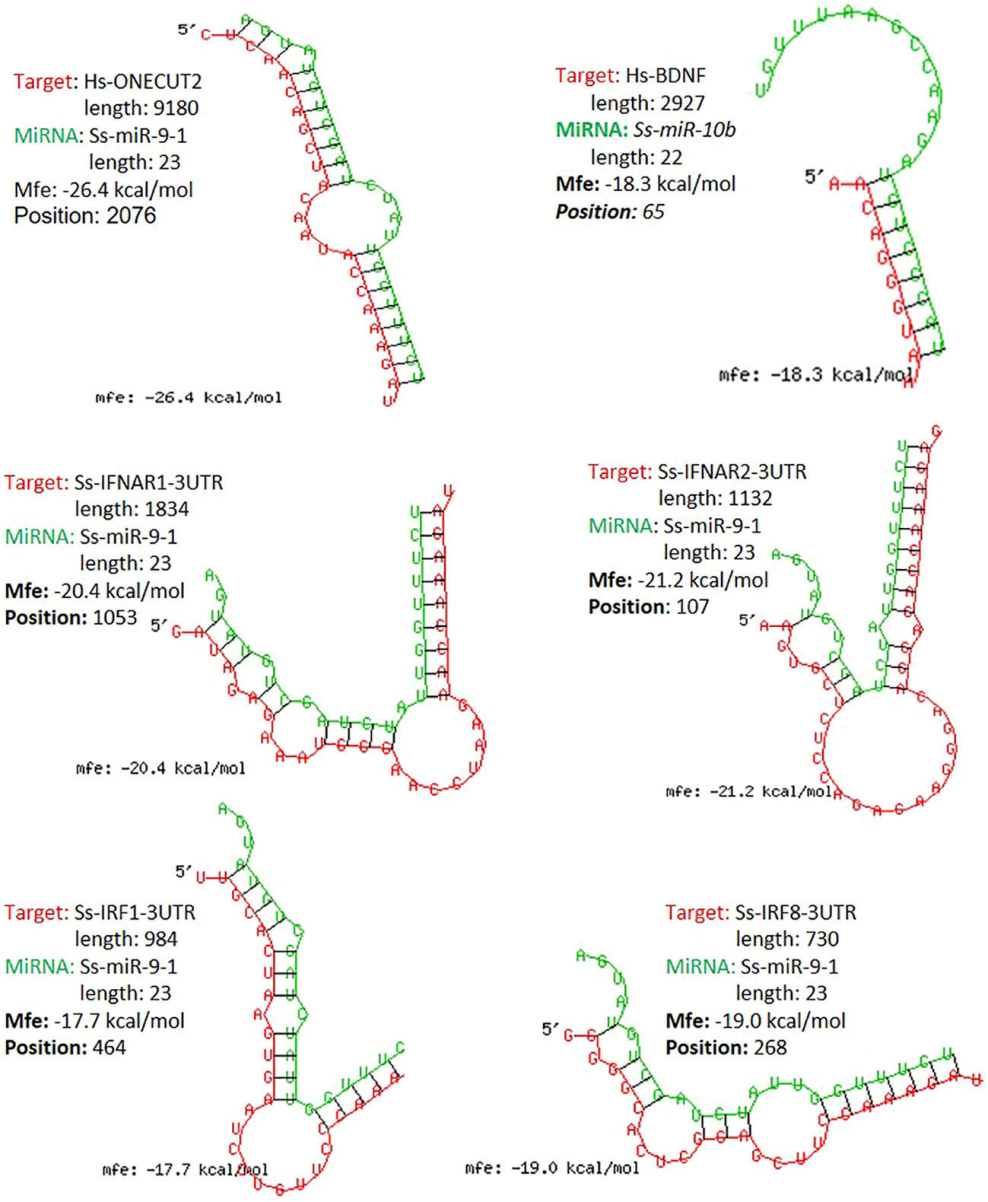
Graphic demonstration of hairpin structures of selected porcine miRNAs and targeted sites on the 3-UTRs of respective human (Hs) or porcine (Ss) transcripts. The selected two miR-9-1 and miR-10b are conserved in both humans and pigs and their targets and effect on human ONECUT2 and BDNF transcripts have been validated in referred studies. Hybridization of individual miRNA with its mRNA targets was performed using RNAhybrid with its accompanying programs RNAcalibrate and RNAeffective as described. No G:U pairing was allowed in the 1-7 seed residues of the 5’-end of miRNAs. The hybridization structures and minimum free energy (Mfe) are given, the thresholds of Mfe was set as -28.0 kcal/mol to reflect the typical Mfe of miRNA (like *let7*) hybridization to mRNA targets. The GenBank Accession numbers of the tested transcripts and all other predicted miRNA-mRNA interactions are provided in the **Supplement Excel Sheet**.

The study functionally examined SEM that were commonly or uniquely coined in the antiviral treatments stimulated by either typical IFN-α1 or unconventional IFN-ω1/-ω5 subtypes (7). **Table 6** lists top ranked SEMs that were shared or unique in PAMs treated by the three subtypes of porcine IFNs. The cross-sample comparison revealed in total about 30 SEMs, which included 10-15 SEM unique for one comparison and 3-5 SEM that were shared by at least two paired comparisons. Two porcine miRNA, ssc-miR-9-1 and ssc-miR-10b, were among the SEM shared by all three IFN treatments compared to the other samples. ssc-miR-9-1 was of Cluster 2 (highly upregulated by MLV/IFN-α1/IFN-ω5 treatments but downregulated in P129/IFNω1 treatments), whereas, ssc-miR-10b was of the Cluster 1 (highly upregulated by MLV/IFN-α1 treatments but downregulated in P129/IFN-ω1/IFN-ω5 treatments). Also ssc-miR-451 and ssc-miR-122-5p, were only notable in IFN-ω1-treatment compared with other treatments. Other SEM were held in Cluster 3 and notable due to their significant upregulation in the P129 (P2) treatment, except ssc-miR-7134-3p, ssc-miR-503, ssc-miR-143-3p and ssc-miR-10a-5p that were upregulated by IFN-α1 as well ssc-miR-503 upregulated by IFN-ω5. In contrast, ssc-miR-122-5p was upregulated by IFN-ω1 but suppressed by IFN-ω5 (**Figure 3** and **Table 6**). We thus examined the existence of target sites on representative PRRSV genome sequences and the 3’-untranslated regions (3’-UTR) of the transcripts for porcine IFNAR1, IFNAR2, JAK1, Tyk2, STAT1/2, and IRFs genes that are key in type I IFN signaling (7, 13, 17). Findings showed that 18 of the SEM (such as ssc-miR-9-1, ssc-miR-19b, and ssc-miR-425-5p containing 2-3 target sites) had stringent target sites along the PRRSV genome sequences (PV), especially within the region of the viral ORF1 that encode viral non-structural proteins (Nsp) that function in catalyzing viral replication and intervening host defense (49). In addition, a majority 24 SEM had stringent target sites on at least one key gene in IFN signaling, and some SEM including ssc-miR-9-1 and ssc-miR-500-5p had 1-2 target sites on the 3’-UTR of several key genes in the IFN signaling pathway (17). Upon further annotation against curated miRNA in macrophage and IFN biology, we observed that near a half of SEM determined in this study were associated with either macrophage activation or IFN signaling pathways, indicated in a cross-study validation of this sRNA transcriptomic analysis (**Table 6** and **Supplemental Excel Sheet**) (16, 17). For example, ssc-miR-9-1 and ssc-miR-10b belong to the miR-9 or miR-10 families respectively that are conserved throughout most mammalian species (30). As miR-9-1 has been reported to regulate both macrophage activation and IFN signaling in studies in humans and mice (17, 50–54), miR-10 seems mostly involved in oncogenesis especially those triggered by viral infections (54–57). Along with several gene targets identified, searching the miRBase database identified over 200 target genes of human miR-9-1 or miR-10b, indicating a potential miRNA-target interaction network underlying the miRNA-mediated epigenetic regulation (48). No study has reported directly about the function of porcine miR-9-1, and one study indicated that porcine miR-10b promoted porcine immature Sertoli cell proliferation by targeting the DAZAP1 gene (58), which is involved in germ cell development. Through searching the miRBase database and evaluation with several miRNA targeting programs, we showed that porcine ssc-miR-9-1 and ssc-miR-10b exerted a similar functional spectrum targeting over 200 gene transcripts. For ssc-miR-9-1, we detected its targeted sites on the 3’-UTR of conservative human (Hs) or porcine (Ss) transcripts, and these porcine transcripts include the IFN receptor (IFNAR1/2) and IFN-regulatory transcription factor (IRF1/8) genes. It was shown that ssc-miR-9-1 and ssc-miR-10b form typical hybridization structures with the target sites of porcine IFNAR1/2, and IRF1/8 genes, and these hybridization structures have the minimum free energy (Mfe) similar to the Mfe of a miRNA hybridizing to verified mRNA targets (**Figure 7**). This provides a good candidate plus those in Cluster 1/3/4 to be experimentally validated for a potential function in IFN-mediated antiviral regulation in PAMs.

**Figure 8:**
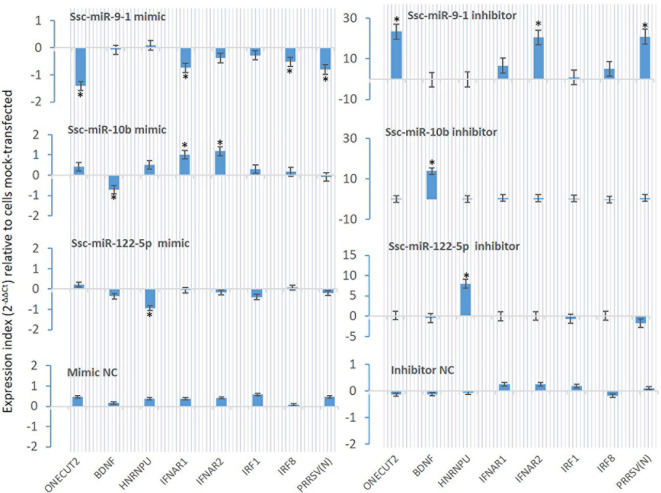
Validation using synthetic siRNA mimics with identical sequences or inhibitors antisense to selected miRNAs. Synthesis of siRNA mimics/inhibitors and transfection of porcine macrophages were performed as described, and gene specific RT-PCR was used to quantify the expression of target genes as labeled under the X-axis. The Gene abbreviation and GenBank Accession numbers of the tested transcripts are listed in **Supplemental Excel Sheets**. *p < 0.05, n = 3 relative to cells mock-transfected.

From **Table 7**, we selected three miRNA that represent most SEM Clusters for experimental validation (**Figure 3**). As all three miRNA were of SEM commonly noted in the different comparisons discussed, ssc-miR-9-1 of Cluster 2 SEM, ssc-miR-10b of Cluster 1 and ssc-miR-122-5p of Cluster 4 showed differentially expression patterns. To functionally validate these miRNA, siRNA mimics of identical sequences or antisense inhibitors to the mature sequences of selected miRNAs were synthesized and transfected into porcine macrophages. As shown in **Figure 8**, transfection of siRNA mimics for ssc-miR-9-1, ssc-miR-10b or ssc-miR-122-5p significantly suppressed the expression of their top-ranked target genes as predicted, i.e., one cut homeobox 2 (ONECUT2) gene by miR-9-1 mimic, the brain-derived neurotrophic factor (BDNF) by miR-10b mimic, and, heterogeneous nuclear ribonucleoprotein U (HNRNPU) by miR-122-5p mimic. Of note, all three genes are highly conserved between pigs and humans. In addition, PAMs transfected with the siRNA mimics for ssc-miR-9-1 were also significantly decreased for the expression of IFNAR1, IRF1 and IRF8, which contain the miRNA target sites predicted on the 3-UTR of their transcripts. In contrast, the suppressive effect was not observed in PAMs transfected with siRNA mimics for ssc-miR-10b and ssc-miR-122-5p, but actually increased IFNAR1/2 expression in PAMs transfected by the mimics for ssc-miR-10b causes unknown. The antisense inhibitors of these miRNA upregulated the expression of ONECUT2, BDNF and HNRNPU, respectively. In addition, the upregulation of IFNAR1, IRF8, especially IFNAR2 was obtained by transfection of inhibitor mimics of ssc-miR-9-1. We also tested if these miRNA mimics contributed to regulation of PRRSV replication in the cells. Data showed that transfection of ssc-miR-9-1 mimics inhibit and the corresponding inhibitor enhance the viral replication measured by copy numbers of the viral ORF7 gene (3, 28). In contrast, the other two miRNA mimics demonstrated little effect on the viral replication, however the inhibitor of ssc-miR-122-5p surprisingly suppressed PRRSV replication, which may correlate to predicted target sites on some porcine ISG genes but will need further verification (**Table 7**). It also indicates that more caution is necessary in examining the potential of the control and inhibitor siRNA oligos, which may exert unexpected effects beyond what they are designed for. It further implies the complexity of the miRNA-mediated network of differential gene expression (47, 48).

**Table 7:** Selected miRNAs for functional validation.

miRNA mimics or inhibitors	Sequence (5'to 3')	Note or Top humangene targets and sites in 3'-UTR*	Comparable predicted sites/positions on 3'-UTRof porcine gene targets
Ssc-miR-9-1 mimic	UCUUUGGUUAUCUAGCUGUAUGA	ONECUT2 (13)	PV(2),IFNARl)(l,IFNAR2(1), IRF(1),IRF8(1)
Ssc-miR-9-1 inhibitor	UCAUACAGCUAGAUAACCAAAGA	(RC to Ssc-miR- 9-1)	IRF9(1)
Ssc-miR-Olb mimic	UACCCUGUAGAACCGAAUUUGU	BDNF (1)	None on tested ISGs
Ssc-miR-Olb	ACAAAUUCGGUUCUACAGGGUA	(RC to Ssc-miR-	STATl(l)
inhibitor		lOb)	
Ssc-miR-122- Sp mimic	UGGAGUGUGACAAUGGUGUUUGU	HNRNPU (1)	None on tested ISGs
Ssc-miR-122- Sp inhbi itor	ACAAACACCAUUGUCACACUCCA	(RC to Ssc-miR- 122-Sp)	IFNAR2(1),JAK(l), IRF2(1),IRF4(1)
MimicNC	UUCUCCGAACGUGUCACGUTT	(No match in miRbase)	None on tested ISGs
Inhibitor NC	CAGUACUUUUGUGUAGUACAA	(No match in miRbase)	None on tested ISGs

*The mature miRNA sequencewas predicted against the UTRs of human mRNA transcripts using TargetScan, and validated in corresponding porcine orthologs using RNAhybrid. BDNF, human brain-derived neurotrophic factor; HNRNPU, heterogeneous nuclear ribonucleoprotein U; NC, negative control; ONECUT2, one cut homeobox 2; RC, reverse complement; Ssc, Sus scrofa.

## Conclusions

Subtype-dependent IFN activity in antiviral and immunomodulatory regulation has been documented in previous studies, but with under-studied molecular mechanisms (7, 13, 17). In this study, we profiled the small RNA transcriptome and differential miRNA response upon antiviral activation by virus infection and IFN stimulation in PAMs that play a critical role in immune regulation in the lung and are targeted by multiple viruses for establishing respiratory infections (3, 4, 15). Findings showed that a majority of sRNA (>98%, including ~90% miRNA) were commonly detected between different antiviral treatments, about 2% sRNA were differentially expressed in the different antiviral treatments by a viral infection or stimulation of different IFN subtypes. Focusing on miRNA, the result profiled 386 porcine miRNA including 331 known and 55 novel miRNA sequences, of which most were ascribed to miRNA families identified from other vertebrate (mammalian) species, which indicates cross-species validation (30). The miRNA compositions were comparable across the different treatments. Detailed comparison showed significantly differentially expressed miRNA (SEM) between the PAMs under the antiviral treatments. The SEM profiles demonstrated that: (1) the pathogenic wild-type and vaccine strains of a PRRSV induced nearly reversed patterns regarding up- or down-regulated SEMs; (2) similar SEM patterns were found among the treatments by the vaccine strain and antiviral IFN-α1/-ω5 subtypes; and (3) the weak antiviral IFN-ω1, showed a suppressive SEM pattern to SEMs upregulated in the three antiviral treatments. The gene ontology and pathway analyses of SEM-targeted genes indicated a broad enrichment in metabolic, inflammatory, antitumor and antiviral pathways, particularly those underlying macrophage activation and IFN signaling (16, 17). Further analyses identified SEMs commonly or uniquely expressed in different treatments, including miR-10b and particularly miR-9-1, which significantly regulate differential antiviral reactions by different IFN subtypes probably *via* epigenetic regulation of several ISGs as well as targeting PRRSV replication (40). In summary, these findings provide a general picture of porcine sRNA composition and pinpoints key SEMs underlying IFN-mediated antiviral regulation in PAMs and interaction with a typical respiratory RNA virus in pigs (49).

## Data availability statement

The datasets presented in this study can be found in online repositories. The names of the repository/repositories and accession number(s) can be found in the article/**Supplementary Material**.

## Author contributions

JL, ES and OA contributed to idea conceptualization, experimental handling, data computation and draft preparation. LM helped in data discussion, manuscript reading, and funding acquisition. YS supervises overall conceptualization, data collection and process, computation, draft writing, and funding acquisition. All authors contributed to the article and approved the submitted version.

## Funding

This work was primarily supported by USDA NIFA Evans-Allen-1013186, NIFA 2018-67016-28313 and NIFA AFRI 2020-67016-31347 to YS, and in part through reagent sharing of NSF-IOS-1831988 to YS.

## Acknowledgments

We thank Jordan Jennings for help with cell and RNA sample preparation.

## Conflict of interest

The authors declare that the research was conducted in the absence of any commercial or financial relationships that could be construed as a potential conflict of interest.

## Publisher’s note

All claims expressed in this article are solely those of the authors and do not necessarily represent those of their affiliated organizations, or those of the publisher, the editors and the reviewers. Any product that may be evaluated in this article, or claim that may be made by its manufacturer, is not guaranteed or endorsed by the publisher.
