# Supplementary material for: Comparative transcriptomics reveals small RNA composition and differential microRNA responses underlying interferon-mediated antiviral regulation in porcine alveolar macrophages
Source: Front Immunol. 2022 Oct 28;13:1016268. doi: 10.3389/fimmu.2022.1016268 (PMC9651005; doi:10.3389/fimmu.2022.1016268)
Supplement: Supplementary file 1 [file DataSheet_1.pdf]

Small RNA

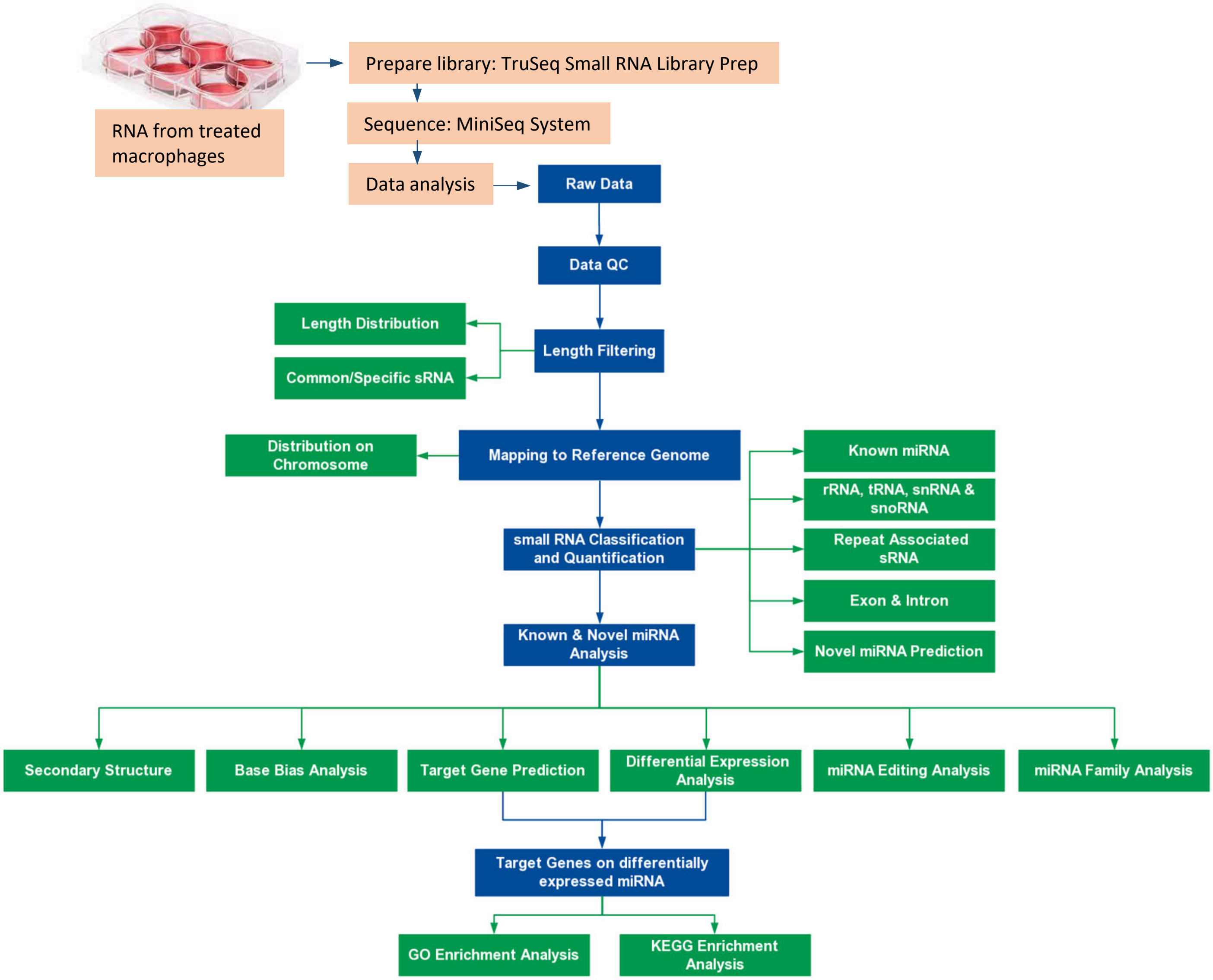

Figure S1. The workflow of whole transcriptomic analysis (WTS) of non-coding small RNA species in porcine macrophages. Primary alveolar macrophages were cultured/treated, and RNA samples at high-quality were obtained for sequencing library preparation and sequencing using an Illumina Sequencer (Steps colored pink). Sequential data analysis is illustrated further with major Steps colored blue and Itemized Steps colored green.

Table S1. Software used in the analysis.

Name	Version	Description	Main Parameter
Bowtie	bowtie-0.12.9	for mapping	-v 0 -k 1
miREvo	miREvo_v1.1	Modify mirdeep2 for known miRNA analysis ; IntegrationmiREvo and mirdeep2 for novel miRNA prediction ; ViennaRNA for mirdeep2 internal call	-i -r -M -m -k -p 10 -g 50000
mirdeep2; ViennaRNA	mirdeep2_0_0_5 ViennaRNA-2.1.1		quantifier.pl -p -m -r -y -g 0 -T 10 default
srna-tools-cli	http://srna-tools.cmp.uea.ac.uk/	for plant TAS prediction	--tool phasing --abundance 3 --pval 0.001 --minsize 20 --maxsize 26 --trrna
RepeatMasker	open-4.0.3	for repeat analysis, based on RepBase18.07 , using trf and irf	-species –nolow -no_is –norna –pa 8
miRanda	miRanda-3.3a	animal target prediction	-sc 140 –en 10 –scale 4 –strict –out
psRobot	psRobot_v1.2	plant target prediction	-s –t –o –p 5
DEseq2	1.12.0	for Biological repeats analysis	padj<0.05
DEGSeq	1.2.2	for no Biological repeats analysis	qvalue<0.01 |log2foldchange|>1
EdgeR	3.2.4	for special circumstances analysis	padj<0.05 |log2foldchange|>1
KOBAS	V3.0	KEGG enrichment	blastx 1e-10; padjust:BH
GOSeq, topGO, hmmscan	Release2.12	enrichmentMethod:Wallenius; padjust:BH	GO Enrichment
Blast	v2.2.28	-evalue 1e-10	protein-protein interactions

Table S2. Summary of the Data Production

Sample*	Reads	Bases	Error rate	Q20	Q30	GC content
P1 (Ctrl)	22637087	1.132G	0.01%	99.43%	98.02%	47.47%
P2 (P129)	25196262	1.260G	0.01%	99.43%	97.94%	47.53%
P3 (MLV)	27045843	1.352G	0.01%	99.27%	97.54%	47.39%
P4 (IFNα1)	22656694	1.133G	0.01%	99.39%	97.79%	47.20%
P5 (IFNω1)	25879946	1.294G	0.01%	99.42%	97.94%	47.02%
P6 (IFNω5)	28480695	1.424G	0.01%	99.16%	97.12%	47.35%

*Note: (1) Sample: Sample ID/Treatment; (2) Reads: Statistics of the original sequence data(3) Bases: Sequence number multiplied the length of the sequence, expressed in gigabase pairs (Gbp); (4) Error rate: Sequencing error rate; (5) Q20: Percentage of bases whose Phred values exceed 20; (6) Q30: Percentage of bases whose Phred values exceed 30; (7) GC content: G and C bases as a percentage of all bases

Table S3. Data filtering summary

Sample*	total reads	N% > 10%	low quality	5' adapter contamin.	3' adapter null or insert null	with ployA/T/G/C
P1 (Ctrl)	22637087 (100.00%)	0 (0.00%)	0 (0.00%)	1652 (0.01%)	136915 (0.60%)	13747 (0.06%)
P2 (P129)	25196262 (100.00%)	2481 (0.01%)	0 (0.00%)	3134 (0.01%)	222008 (0.88%)	24013 (0.10%)
P3 (MLV)	27045843 (100.00%)	0 (0.00%)	0 (0.00%)	2176 (0.01%)	304746 (1.13%)	17174 (0.06%)
P4 (IFNα1)	22656694 (100.00%)	0 (0.00%)	0 (0.00%)	1365 (0.01%)	107177 (0.47%)	12344 (0.05%)
P5 (IFNω1)	25879946 (100.00%)	2461 (0.01%)	0 (0.00%)	1825 (0.01%)	171338 (0.66%)	12749 (0.05%)
P6 (IFNω5)	28480695 (100.00%)	230 (0.00%)	0 (0.00%)	1615 (0.01%)	136339 (0.48%)	15195 (0.05%)

*Note: (1) Sample: Sample ID/Treatment; (2) Total Reads: Total sequenced reads; (3) N% > 10%: Percentage of reads with N > 10%; (4) Low Quality: Percentage of low quality reads; (5) 5' Adapter Contamination: Percentage of reads with 5' adapter contamination; (6) 3' Adapter or Insert Missing: Percentage of reads without a 3' adapter or insert; (7) With Poly(A)/(T)/(G)/(C) : Percentage of reads with poly(A), poly(T), poly(G), or poly(C) tails; (8) Clean Reads: Total clean reads followed by clean reads as a percentage of raw reads

Table S4. Statistics of mapping results on either positive (+) or negative (-) DNA strands

**Sample***	**Total sRNA**	**Mapped sRNA**	**"+" Mapped sRNA**	**"-" Mapped sRNA**
P1 (Ctrl)	21648455 (100.00%)	19760012 (91.28%)	18342559 (84.73%)	1417453 (6.55%)
P2 (P129)	24486844 (100.00%)	21676078 (88.52%)	20108464 (82.12%)	1567614 (6.40%)
P3 (MLV)	26139112 (100.00%)	23592435 (90.26%)	21917884 (83.85%)	1674551 (6.41%)
P4 (IFNα1)	22282175 (100.00%)	20078270 (90.11%)	18683796 (83.85%)	1394474 (6.26%)
P5 (IFNω1)	25470808 (100.00%)	22662197 (88.97%)	21224933 (83.33%)	1437264 (5.64%)
P6 (IFNω5)	27776163 (100.00%)	25001252 (90.01%)	23253493 (83.72%)	1747759 (6.29%)

*Note: (1) Sample: Sample ID/Treatment; (2) Total sRNA: Number of total sRNA after length filtering; (3) Mapped sRNA: Number and percentage of sRNA mapped to genome; (4) + Mapped sRNA: Number and percentage of mapped sRNA in the same direction (sense) as the genome; (5) – Mapped sRNA: Number and percentage of mapped sRNA in the opposite direction (antisense) to the genome

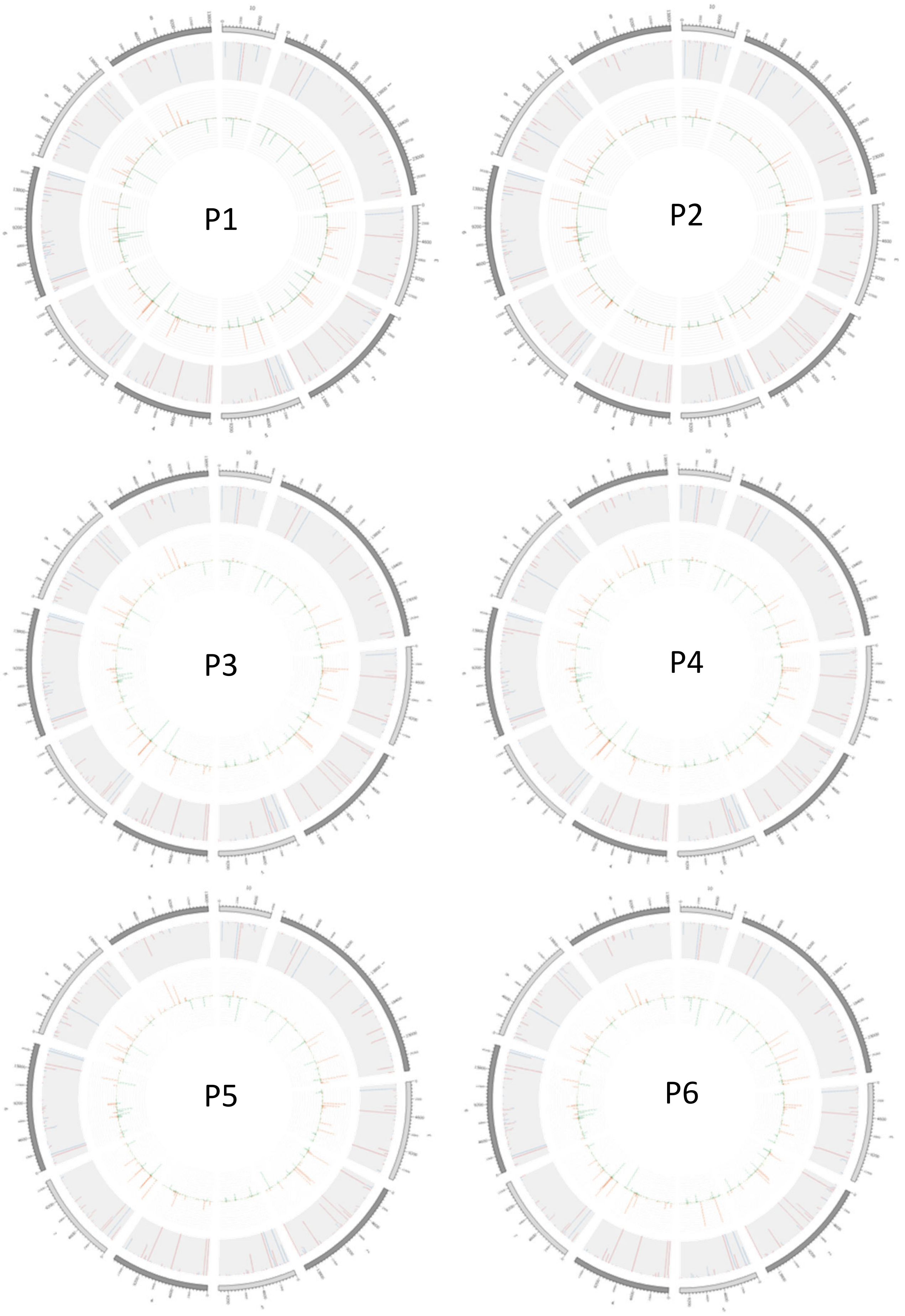

Figure S2. Circos diagrams showing sRNA reads distribution per chromosome. The longest 10 contigs or scaffolds of current swine reference genome were chosen for analysis. The chromosome is shown as the outer circle. Grey background in the middle area shows the distribution of 10,000 reads on the chromosome. Red represents the number of sRNAs on the sense strand of the chromosome, and blue represents the number of sRNAs on the antisense strand. All reads are shown in the center area of the circle. Yellow represents the number of sRNAs on the sense strand of the chromosome, and green represents the number of sRNAs on the antisense strand.

Total sRNA

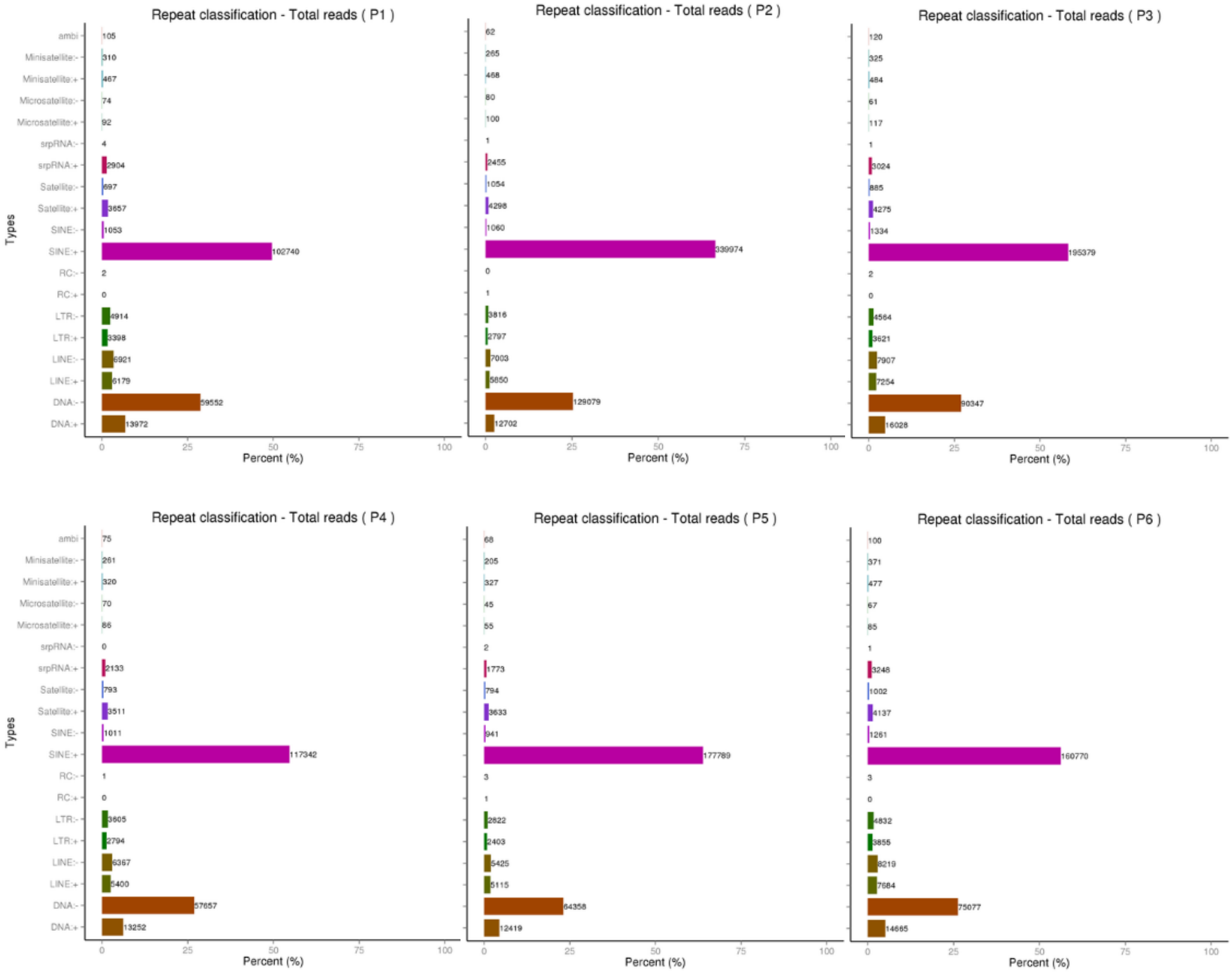

Figure S3A. Total sRNA aligned to different repeat sequences. To avoid redundancy, sRNA were aligned with annotated/predicted repeating sequences and repetitive reads of sRNA were polished for counting accuracy. Statistics on the various repeats of total sRNAs (A) and uniquely expressed in each sample were calculated and shown.

Uniquely expressed sRNA in each sample

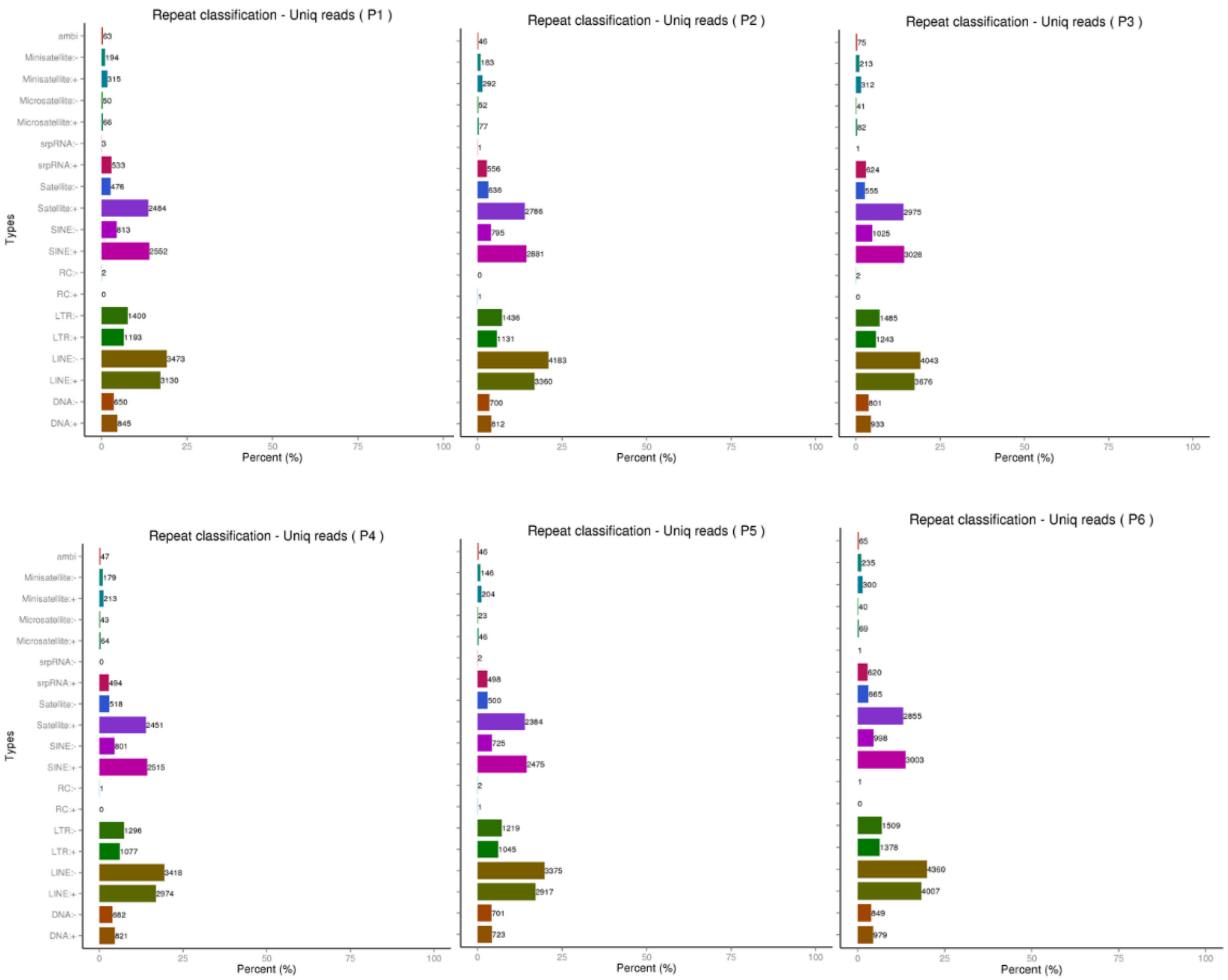

Figure S3B. Uniquely expressed sRNA aligned to different repeat sequences. To avoid redundancy, sRNA were aligned with annotated/predicted repeating sequences and repetitive reads of sRNA were polished for counting accuracy. Statistics on the various repeats of total sRNAs (A) and uniquely expressed in each sample (B) were calculated and shown.

Table S5. sRNAs mapped to exon and intron

Types	P1 (Ctrl)	P2 (P129)	P3 (MLV)	P4 (IFNα1)	P5 (IFNω1)	P6 (IFNω5)
exon	677477	1156755	952265	756532	809800	952570
exon:+	420786	824378	606062	504336	593494	599770
exon:-	256691	332377	346203	252196	216306	352800
intron	317513	540719	432199	316655	344670	415429
intron:+	233254	387720	322917	228469	252738	313408
intron:-	84259	152999	109282	88186	91932	102021

Note: (1) Types: Types of exon and intron, +/-, positive or negative direction; (2) Columns 2-n+1 show each corresponding sample's results

Note: Exon and Intron Alignment. The sequences of sRNA sequencing may have degraded fragments of mRNA. This part of the analysis is to annotate the sequencing reads as much as possible. On another hand, it is to remove the sequences from these genes before the new miRNA predicted. As these regions might be the product of degraded fragments. These regions can predict relatively few miRNAs compared to the gene intergenic region. In order to eliminate interference, this part of the reads is currently taken directly.

Table S6. Cross-species comparison of miRNA families identified*

Selected miRNA family /organism species	*mir-490*	*mir-191*	*mir-134*	*mir-1343*	*mir-486*	*mir-29*	*mir-503*	*mir-322*	*mir-224*	*Let-7*	(146 more)
Sus scrofa	+	+	-	+	+	+	+	+	+	+	...
Homo sapiens	+	+	+	+	+	+	+	+	+	+	...
Pan troglodytes	+	+	+	-	+	+	+	+	+	+	...
Bos taurus	+	+	+	+	+	+	+	+	+	+	...
Ovis aries	-	+	+	-	-	+	-	-	-	+	...
Mus musculus	+	+	+	-	+	+	+	+	+	+	...
Rattus norvegicus	+	+	+	-	+	+	+	+	+	+	...
Gallus gallus	+	-	-	-	-	+	-	-	-	+	...
Xenopus tropicalis	-	+	-	-	-	+	-	-	-	+	...
Xenopus laevis	-	-	-	-	-	-	-	-	-	-	...
Salmo salar	-	-	-	-	-	+	-	-	-	+	...
Danio rerio	-	-	-	-	-	+	-	-	-	+	...
Drosophila melanogaster	-	-	-	-	-	+	-	-	-	+	...
Arabidopsis thaliana	-	-	-	-	-	-	-	-	-	-	...

*Note: In general, miRNAs in same family conserve at sites 2-7 nt of the seed sequence to regulate a target gene in a specific biological pathways. Sequence similarity comparison is the most straightforward method to annotate miRNA families. This table explores the occurrence of known miRNA and novel miRNA families, identified from samples of the targeted and comparative species. "+", the miRNA family exists in this species, and "-", the miRNA family does not exist in this species.

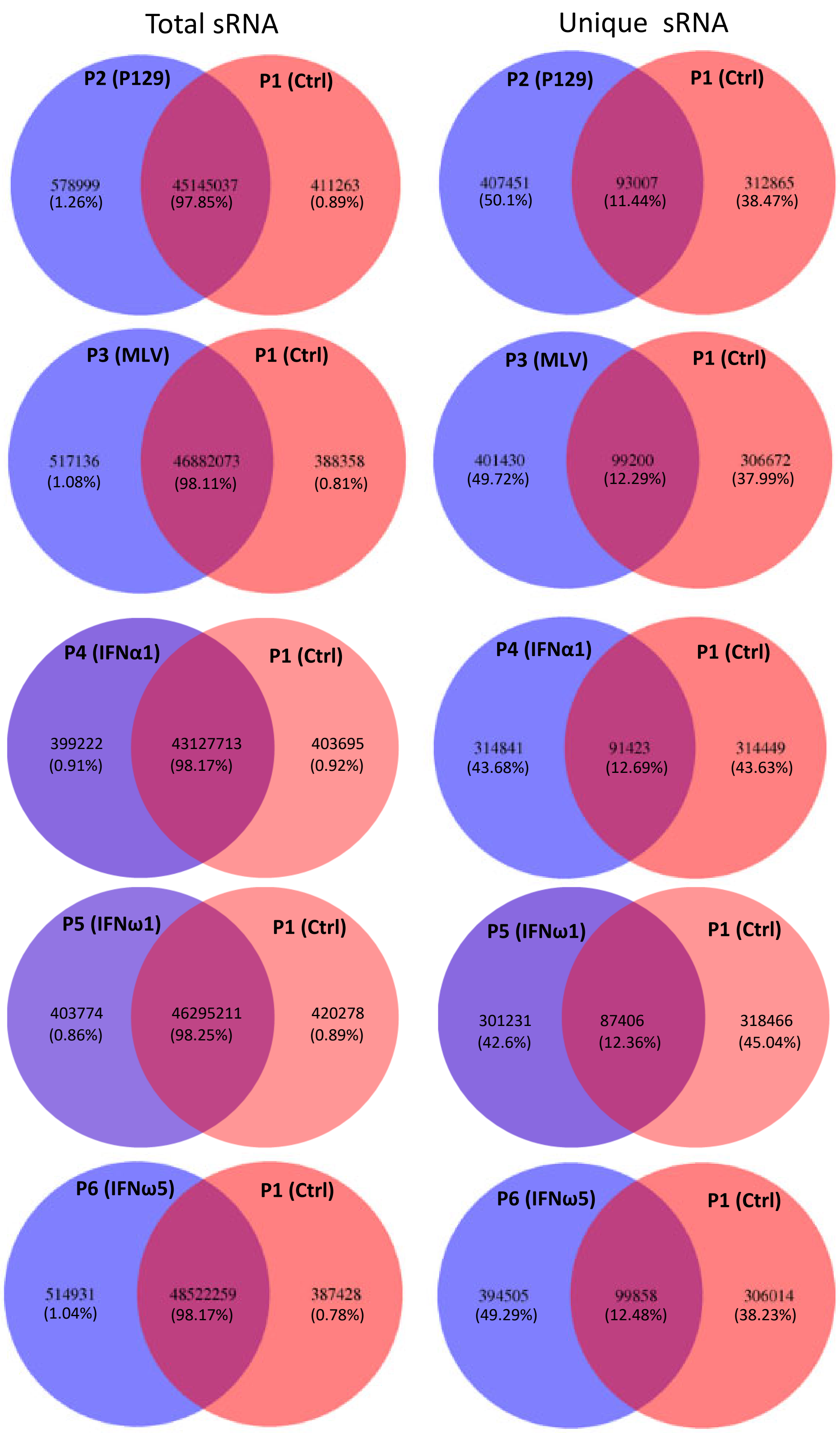

Figure S4. Venn diagrams showing **c**ommon and specific reads between samples of different treatments with the control. The left Column is for total sRNA and the right for unique sRNA common to both samples or specific to each sample compared. Both qualified sRNA read numbers (top) and percent (bottom) are provided.

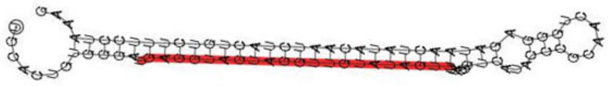

Figure S5. The secondary structure of the known miRNAs (exemplified with ssc-let-7a) on partial schematic matches. The entire sequence is an miRNA precursor, the red section is the mature sequence.

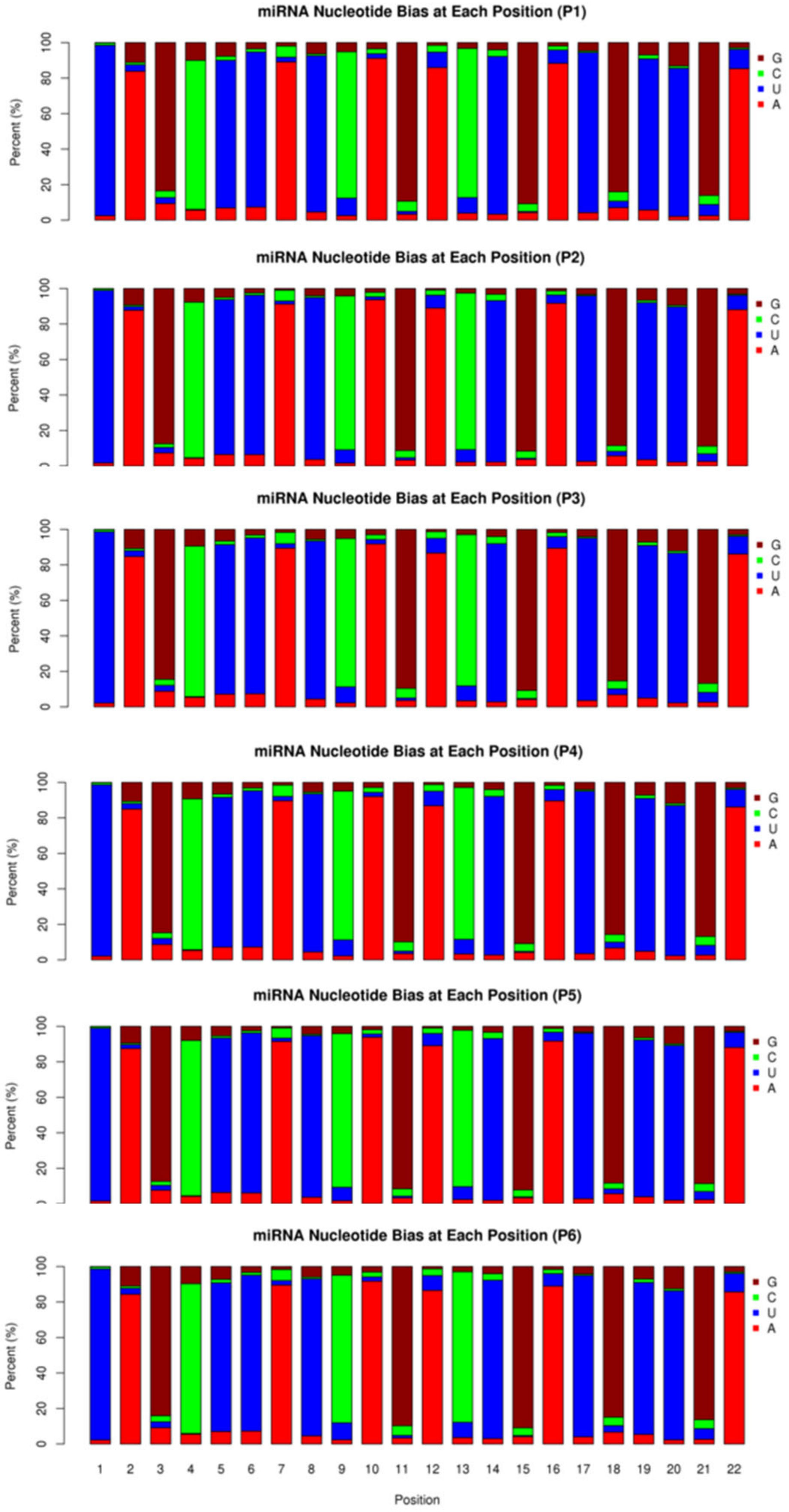

Figure S6. miRNA nucleotide bias at each position. The X axis shown each position of miRNA nucleotide, The Y axis shown the percentage. Data showing near identical nucleotide bias at each position of miRNA species from all samples, indicating general comparability among samples but relative difference in expression levels.

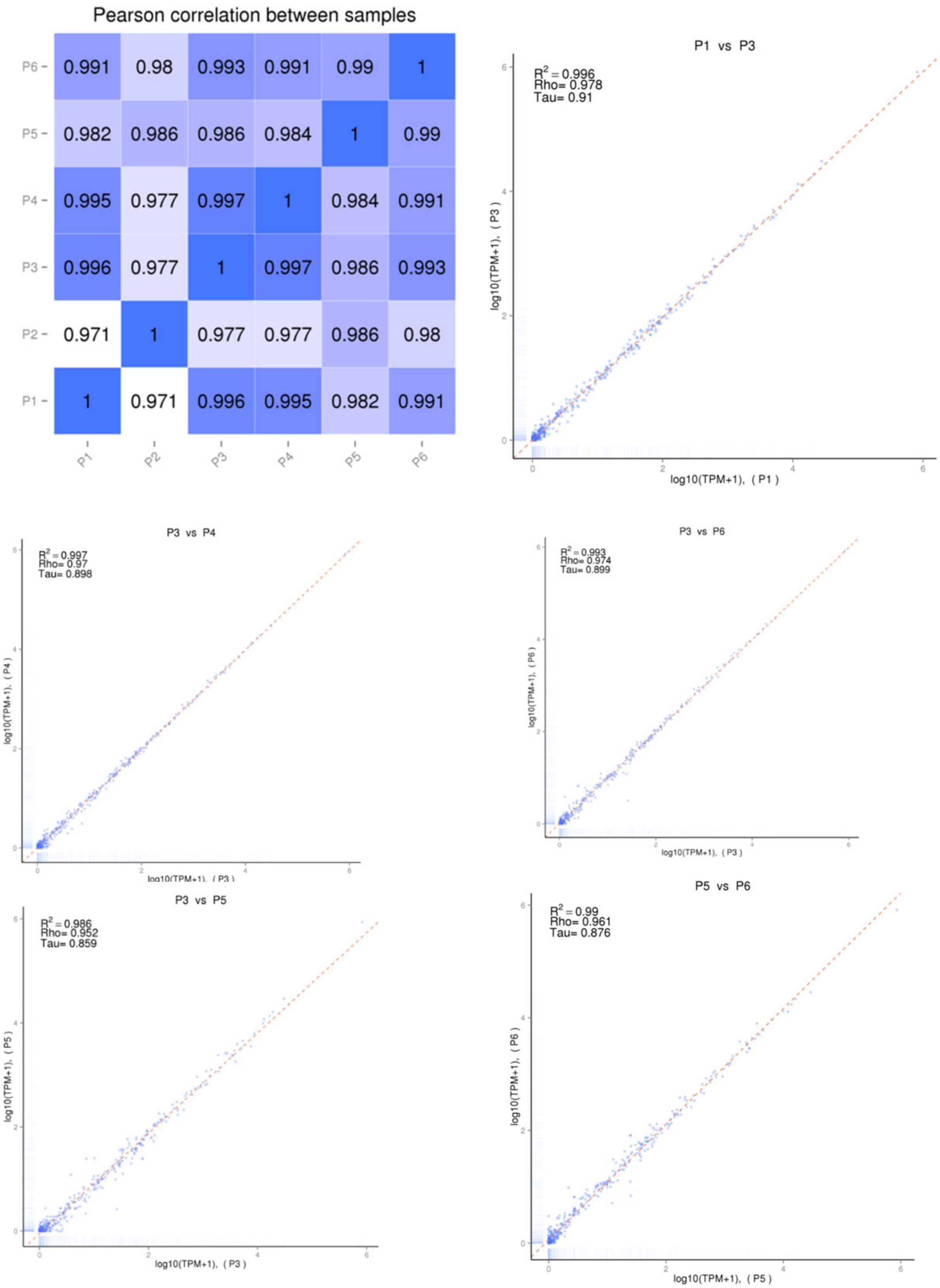

Figure S7. RNA-Seq correlation analysis between samples.The x axis and y axis represented the $\log_{10}$(TPM+1) and correlation coefficients obtained using diffident algorithms as indicated (R^2: pearson RSQ; Rho: spearman coefficient of association； Tau: kendall-tau coefficient of association). The closer correlation coefficient is to 1, the higher the more similar between the samples. Encode suggests that the square of the Pearson correlation coefficient (R^2or RSQ) should be larger than 0.92 under ideal experiment conditions.

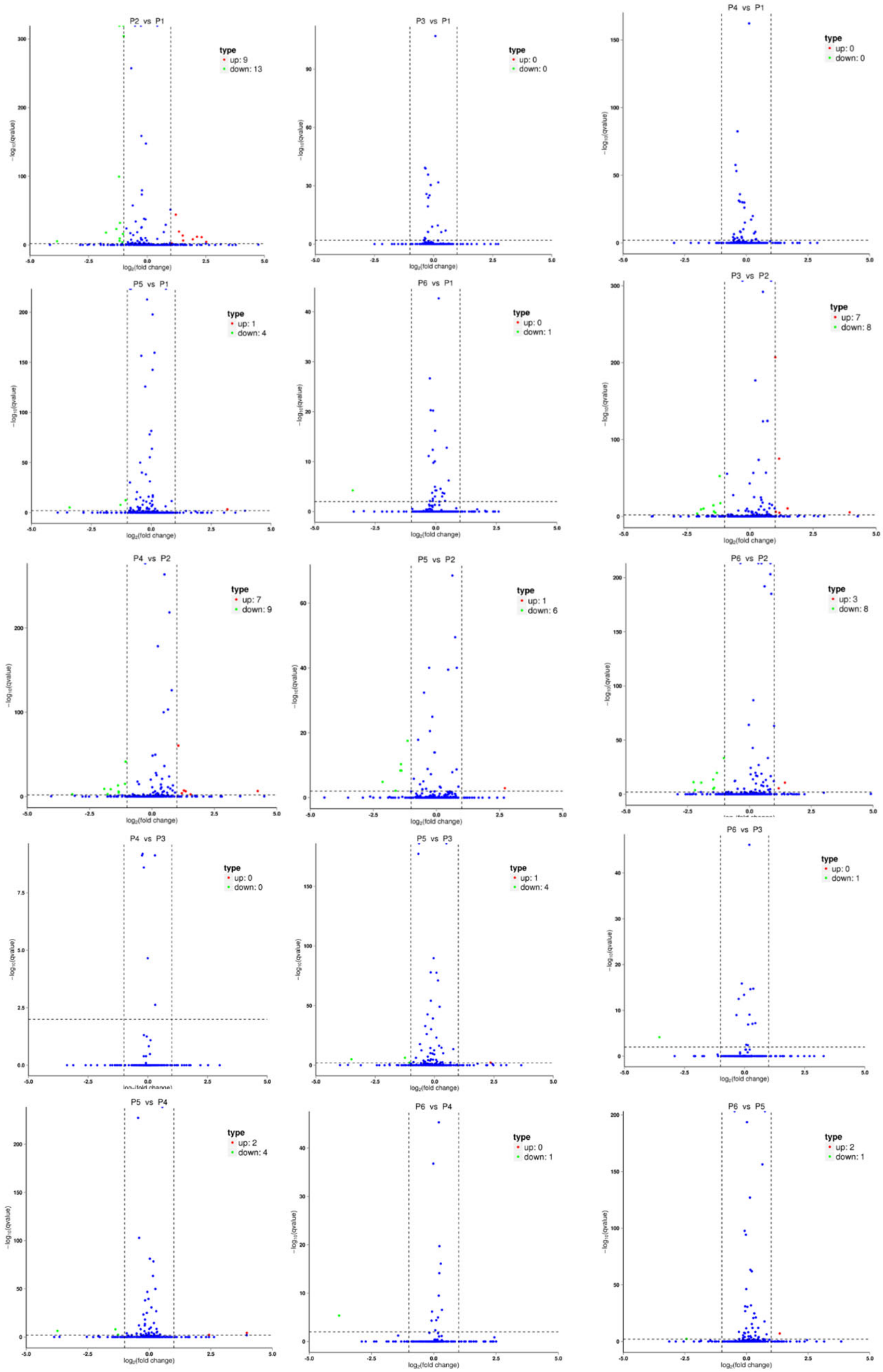

Figure S8. Volcano Plots to show Differential expression of differentially expressed miRNA (DEM) in compared samples as indicated. The X-axis shows the fold change in miRNA expression between different samples, and the Y-axis shows the statistical significance of the difference. Statistically significant difference (|log2(FoldChange)| > 1 and q-value < 0.01) is represented by red or green dots for up- or down-regulated miRNA, respectively.

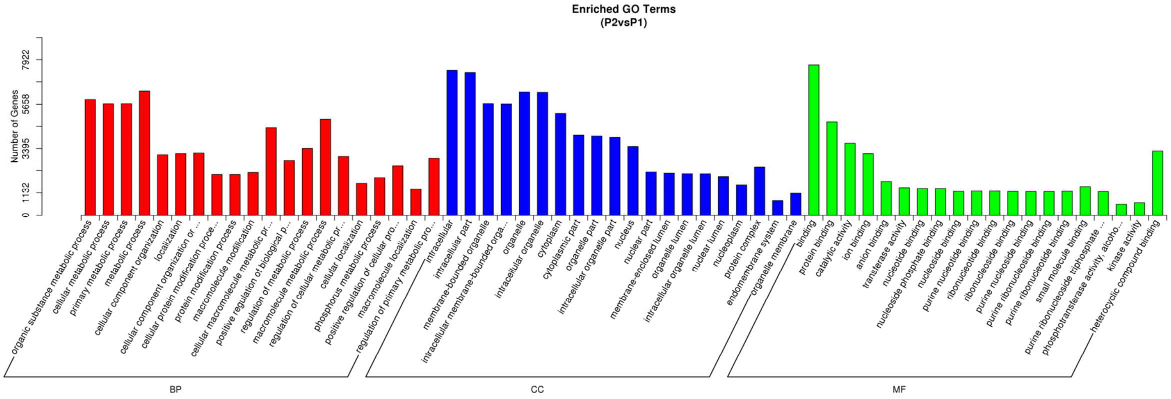
Enriched GO Terms
(P2vsP1)
Number of Genes
0
1132
3395
5658
7922
BP
CC
MF

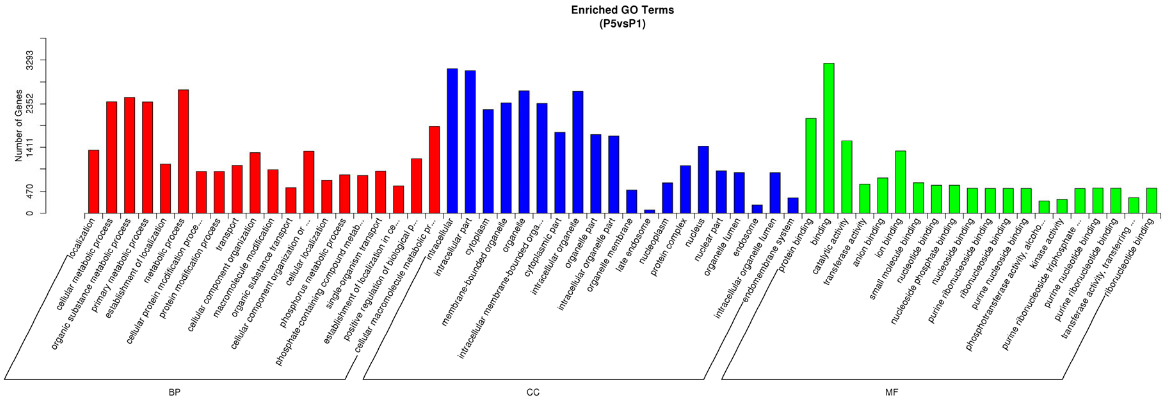
Enriched GO Terms
(P5vsP1)
Number of Genes
0
470
1411
2352
3293
BP
CC
MF

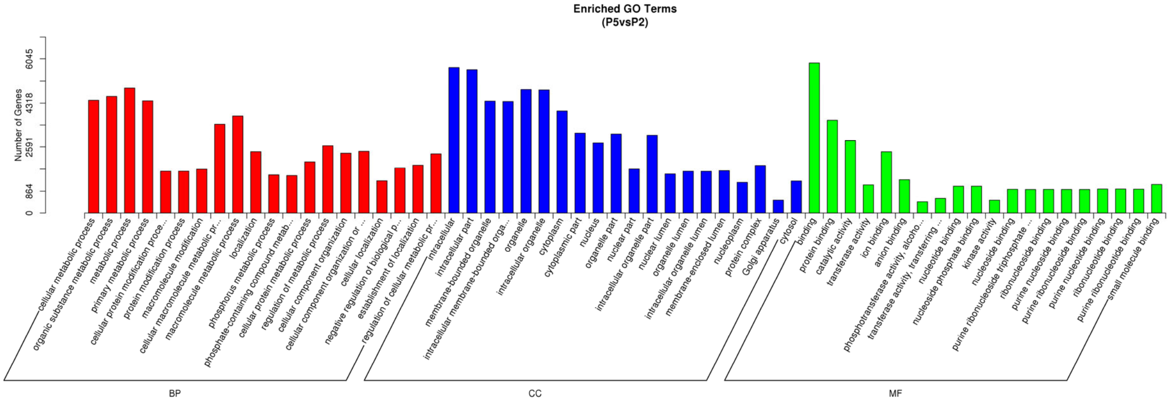
Enriched GO Terms
(P5vsP2)
Number of Genes
0
864
2591
4318
6045
BP
CC
MF

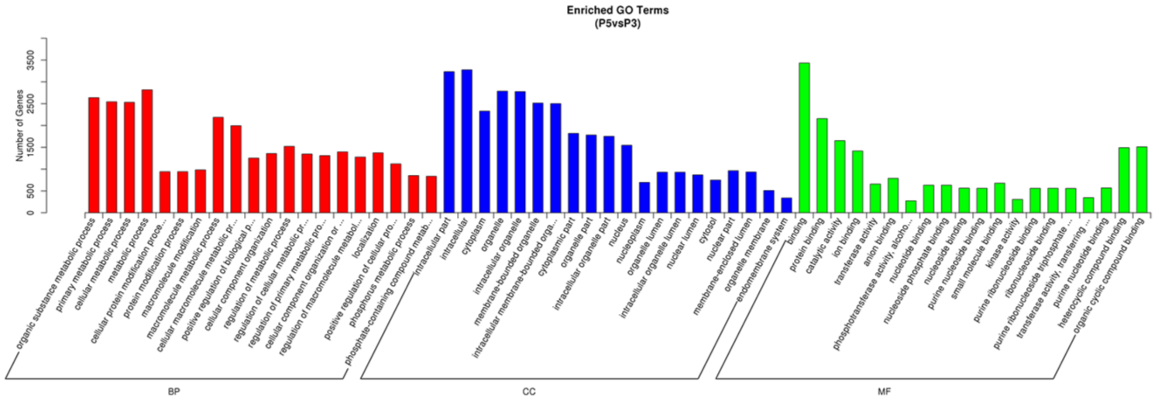
Enriched GO Terms
(P5vsP3)
Number of Genes
0
500
1500
2500
3500
BP
CC
MF

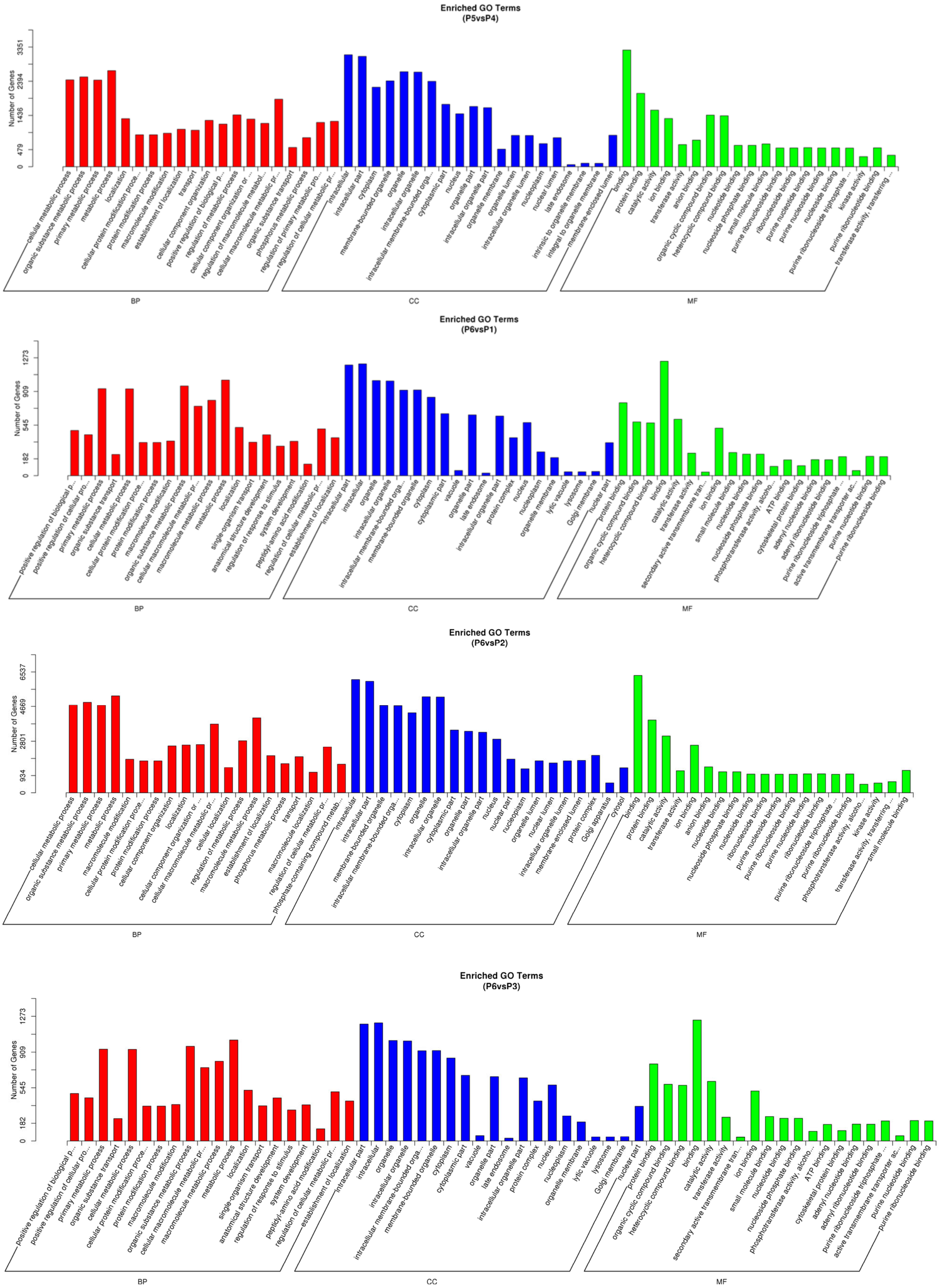

Figure S9. Gene Ontology (GO) analysis to unify miRNA-targeted genes in three main branches: cellular compartment (CC), molecular function (MF) and biological process (BP). Showing are GO terms with p-adj < 0.05 of significant enrichment in selected comparison between samples. Wallenius non-central hyper-geometric distribution was used to find out significantly enriched GO terms in target gene candidates relative to the reference gene background (http://www.geneontology.org/).

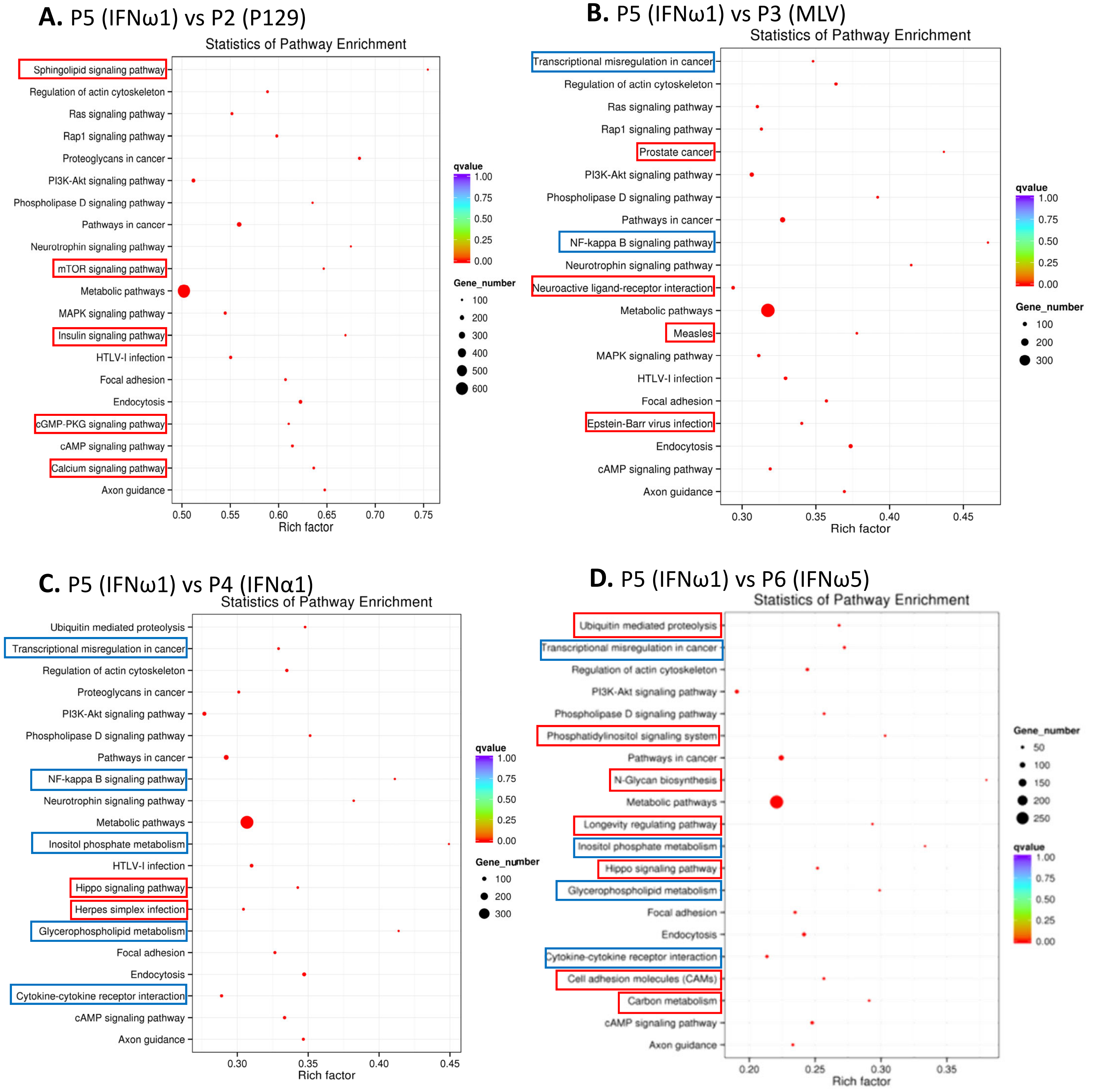

Figure S10. Scatter plots of pathway enrichment analysis of genes targeted by significantly differentially expressed miRNAs. Pathway enrichment analysis were conducted based on the Kyoto Encyclopedia of Genes and Genomes (KEGG) database. The Y-Axis lists significantly enriched pathways and X-Axis shows the Rich factor. Dot size represents the number of miRNA-targeted genes and the color indicates the scale of q-value. Pathways framed indicated unique pathways enriched in each compared pair only (Red frame) or shared by different compared pairs (Blue frame).
